# The associations of dietary patterns with depressive and anxiety symptoms: a prospective study

**DOI:** 10.1186/s12916-023-03019-x

**Published:** 2023-08-15

**Authors:** Han Chen, Zhi Cao, Yabing Hou, Hongxi Yang, Xiaohe Wang, Chenjie Xu

**Affiliations:** 1 School of Public Health, Hangzhou Normal University, Hangzhou, China; 2 Hangzhou International Urbanology Research Center & Center for Urban Governance Studies, Hangzhou, China; 3 School of Public Health, Zhejiang University School of Medicine, Hangzhou, China; 4 Yanjing Medical College, Capital Medical University, Beijing, China; 5 Department of Bioinformatics, School of Basic Medical Sciences, Tianjin Medical University, Tianjin, China

**Keywords:** Depression, Anxiety, Dietary pattern, Reduced rank regression

## Abstract

**Background:**

Diet is increasingly recognized as an important risk factor for mental health. However, evidence regarding the association between diet pattern and depressive and anxiety symptoms is limited. We aimed to investigate the associations of dietary patterns characterized by a set of nutrients of interest with depressive and anxiety symptoms.

**Methods:**

The analyses included a total of 126,819 participants in the UK Biobank who had completed at least two dietary questionnaires. Dietary data were obtained through 24-h dietary assessment at baseline between 2006 and 2010 and four rounds of online follow-ups between 2011 and 2012. Reduced rank regression was applied to derive dietary patterns (DPs) explaining variability in energy density, free sugars, saturated fat, and fiber intakes. Depressive and anxiety symptoms were measured by the Patient Health Questionnaire-9 and General Anxiety Disorder-7 between 2016 and 2017, respectively. Logistic regression models were performed to investigate the associations between dietary patterns and depressive and anxiety symptoms.

**Results:**

During a mean follow-up of 7.6 years, 2746 cases of depressive symptoms and 2202 cases of anxiety symptoms were recorded. Three major DPs were derived, explaining 74% of the variation in nutrients hypothesized to be related to depressive and anxiety symptoms. DP1 was characterized by high intakes of chocolate, confectionery, butter, and low vegetable/fruit intakes. Compared to the lowest quintile of DP1, the odds ratio (95% confidence interval) of depressive symptoms for Q2–Q5 was 0.82 (0.72–0.93), 0.86 (0.76–0.98), 1.02 (0.90–1.15), and 1.17 (1.03–1.32), respectively. Compared to the lowest quintile of DP1, the odds ratio (95% CI) of anxiety symptoms for Q2–Q5 was 0.84 (0.73–0.97), 0.91 (0.79–1.05), 1.01 (0.88–1.15), and 1.18 (1.03–1.35), respectively. DP2 featured high intakes of sugar-sweetened beverages, added sugars, and low intakes of butter/cheese but showed no significant links to depressive or anxiety symptoms. DP3 was characterized by high butter and milk desserts and low alcohol/bread intakes. Compared to the lowest quintile of DP3, the odds ratio (95% CI) of depressive symptoms for Q2–Q5 was 0.90 (0.79–1.01), 1.00 (0.88–1.13), 1.06 (0.94–1.20), and 1.17 (1.03–1.32), respectively. Compared to the lowest quintile of DP3, the odds ratio (95% CI) of anxiety symptoms for Q2–Q5 was 0.90 (0.78–1.04), 1.05 (0.91–1.20), 1.02 (0.89–1.17), and 1.21 (1.05–1.38), respectively.

**Conclusions:**

A DP characterized by high intakes of chocolate and confectionery, butter, high-fat cheese, added sugars, along with low intakes of fresh fruit and vegetables, is associated with a higher risk of depressive and anxiety symptoms.

**Supplementary Information:**

The online version contains supplementary material available at 10.1186/s12916-023-03019-x.

## Background

Common mental disorders are estimated to affect more than 970 million people worldwide, with depressive and anxiety disorders being leading causes of disease burden and major contributors to disability [1]. A growing body of evidence showed that depression and anxiety were associated with higher morbidity and suicide risks [2, 3]. Diet habit has been increasingly examined as a modifiable risk factor in the etiology of these mental disorders [4–6].

Previous studies have demonstrated an increased risk of depressive or anxiety symptoms associated with high intakes of single nutrients, such as saturated fat [7], free sugars [8], or low intakes of fiber [9]. There is also some evidence focusing on the relationships between food items and common mental disorders, such as sugar-sweetened beverages [10], meat [11], fruit, vegetables [12], and fish [13]. However, the effect of individual nutrient or food may not be sufficient to reflect the health effects of a whole diet pattern, since foods are always consumed in combination. So far, the synergistic effects of various food groups or nutrients on depressive and anxiety symptoms are largely unknown.

Reduced rank regression (RRR) is a data-driven statistical method to characterize major dietary patterns (DPs), which could explain the maximum amount of variation of nutrients based on a priori hypotheses of the pathophysiology of disease [14]. The RRR, a classification method of diet pattern, may build a more robust link between dietary patterns and health outcomes [15]. Therefore, the aim of this study was to investigate the associations between dietary patterns derived by RRR and depressive and anxiety symptoms.

## Methods

### Study population

UK Biobank is a prospective cohort study involving > 500,000 participants aged 37–73 years across the UK from 2006 to 2010. It was approved by the NHS National Research Ethics Service (Ref. 11/NW/0382) and all participants gave written informed consent at study entry. Details of the study design and data collection have been described previously [16]. From 2016 to 2017, 157,366 participants completed an online mental health questionnaire to record the symptoms of mental disorders [17].

**Fig. 1:**
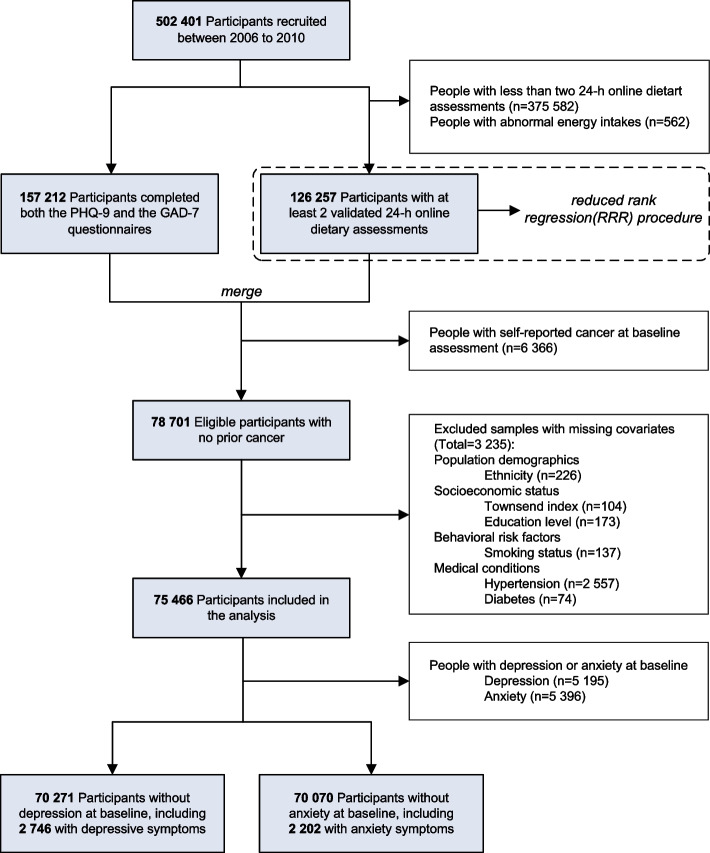
Flow chart of the study

Our analyses were restricted to the 157,212 participants who had information on depression and anxiety measures, in which 85,067 completed at least two rounds of dietary questionnaires. Participants were excluded because of abnormal energy intake (men with < 800 or > 4200 kcal/day, and women with < 600 or > 3500 kcal/day) [18]; existing history of cancer at baseline assessment; and missing information on covariates. Depression and anxiety events that occurred before or at baseline were identified using antidepressant use and linked hospital admissions data. Thus, we further excluded 5195 and 5396 participants who were separately diagnosed with depressive and anxiety symptoms, leaving 70,271 and 70,070 participants without depression or anxiety at baseline included in the final study, respectively. A flow chart of this study can be seen in Fig. 1.

### Dietary assessment

A web-based 24 h dietary assessment tool named Oxford WebQ was used to collect information on dietary intakes [19], including the frequency of 206 common foods and 32 types of beverages consumed during the previous day. The Oxford WebQ is a suitable tool for repeated measurements in large-scale prospective studies, such as the UK Biobank study and the Million Women Study. Its reliability and validity have been confirmed through comparison with an interviewer-administered 24-h dietary recall method [19]. Participants with validated e-mail addresses were invited to respond to the Oxford WebQ questionnaire at baseline assessment and additionally followed up to four times between February 2011 and June 2012 at 3–4 monthly intervals. We calculated the average intake of nutrients and food groups across the five rounds of dietary questionnaires. Based on the methodology described previously [20], food intakes were classified into 50 groups based on the similarity in nutritional content or culinary usage. Total energy and nutrient intake data were generated based on the UK Nutrient Databank food composition tables [21]. Energy density, saturated fatty acids, fiber density, and free sugars were selected as the nutrients of interest, indicating their role in the development of depression and anxiety [5, 22]. Saturated fatty acids and free sugars in % total Energy (%E) were calculated by multiplying the intake in g/day with 25 kJ/g or 17 kJ/g. Fiber intake was estimated using the Englyst method [23].

### Ascertainment of outcomes

The ascertainment of depression was based on the Patient Health Questionnaire-9 (PHQ-9), which is a nine-item questionnaire for depression screening. The PHQ-9 scores the 9 DSM-IV criteria separately as “0” (not at all) to “3” (nearly every day), yielding an overall depression severity score between 0 and 27 [24]. A cut-off score of 10 or above is recommended to identify the probable cases of major depression [25]. Meanwhile, anxiety was measured by the General Anxiety Disorder-7 (GAD-7). GAD-7 is a 7-item measure for general anxiety disorder screening and severity assessment [26]. It is also a scale rating 0–3, with a total sum score ranging from 0 to 21. A cut-off score of 10 or above is recommended to identify the presence of anxiety disorders [27]. In the study, follow-up time was calculated from baseline assessments (2006–2010) to completion of PHQ-9 and GAD-7 questionnaires (2016–2017), serving as start and endpoints for participants respectively.

### Ascertainment of covariates

Covariates were selected based on a prior-defined directed acyclic graph (Additional file 1: Fig. S1). Our study finally included age (calculated at baseline assessment centers), sex (female, male), ethnicity (white, others), Townsend deprivation index, education level (college qualification, other qualification, none qualification), self-reported smoking status (never, previous, current), physical activity (low, moderate, high, missing data), history of hypertension (yes/no), history of diabetes (yes/no), and history of cardiovascular disease (yes/no). Townsend deprivation index represents the level of deprivation on the basis of post codes. It was derived from aggregated data of unemployment, car ownership, house ownership, and household overcrowding, with higher scores indicating higher deprivation [28]. Physical activity was collected from an adapted version of the short International Physical Activity Questionnaire (IPAQ), and the total metabolic equivalent of task (MET) in a week was categorized into high (≥ 3000 MET-minutes per week), moderate (≥ 600 and < 3000 MET-minutes per week), and low (< 600 MET-minutes per week) [29]. Prevalence of hypertension, diabetes, and cardiovascular disease was based on self-report data of a doctor diagnosis and verified during the face-to-face interview.

### Statistical analyses

#### Identification of dietary patterns

Dietary patterns (DPs) were derived through the use of reduced rank regression (RRR), a data-driven statistical method that employs a priori knowledge to select nutrients hypothesized to be relevant to disease risk. RRR identifies linear functions of food groups that explain as much variation as possible in the pre-selected nutrient response variables, resulting in dietary patterns associated with the nutrients of interest based on the initial hypothesis [14]. Energy density, saturated fatty acids, free sugars, and fiber density were used as response variables in the RRR model. Dietary pattern scores were calculated for each participant using reduced rank regression, assigning individuals a *z*-score representing adherence to each identified pattern. Participants were then categorized into quintile groups based on DP *z*-scores, creating exposure groups with increasing levels of adherence to each dietary pattern. Factor loadings of food intake were positively correlated to DP *z*-score. A higher factor loading indicates a greater contribution of that food group to the DP. The associations between dietary patterns and nutrient-response variables were evaluated by correlation coefficients (Additional file 1: Table S1). We retained dietary patterns with explained variance > 10% for subsequent analyses.

#### Associations of DPs with depressive and anxiety symptoms

Dietary patterns identified from RRR were categorized into quintiles, with the lowest quintile (Quintile 1) as the referent. Logistic regression models were used to estimate odds ratios (ORs) and 95% CIs (confidence intervals) for depressive and anxiety symptoms, with adjustment for age, sex, ethnicity, Townsend deprivation index, education level, smoking status, physical activity, history of hypertension, history of diabetes, and history of cardiovascular disease.

Potential non-linear associations between dietary patterns and depression and anxiety were tested using restricted cubic splines (three to five knots were chosen based on Akaike’s information criterion [AIC], and 95% of the DP score distribution [30]) fitted on the logistic regression models with multivariable adjustment, and the 5% quantile of DP scores were used as the reference.

### Sensitivity analyses

Several sensitivity analyses were conducted to test the robustness of our findings. First, to explore potential bias related to the random variation in individual intakes, we repeated the RRR analysis with participants completing ≥ 1 (*N* = 188,909), ≥ 3 (*N* = 71,270), ≥ 4 (*N* = 32,850) or ≥ 5 (*N* = 5195) dietary assessments. Second, to investigate the influence of missing variables, multiple imputation by chained equations with 10 imputations were used to impute all missing covariates [31]. Third, logistic regression models were further stratified according to age groups (37–50, 50–60, 60–73 years), sex (female, male), smoking status (never, previous, current), and physical activity (inactive, active), in which *P* values for interaction were evaluated using interaction terms and likelihood ratio tests. Fourth, we inputted energy density, saturated fatty acids, fiber density, and free sugars separately as single response variables in the RRR model and repeated the main analyses, aiming to isolate the effect of nutrients in DPs to shed light on the not fully elucidated links between DPs and depression and anxiety. Considering RRR as a multivariate model, the results were from general linear regression for a single response variable scenario. Fifth, we conducted multivariable Cox proportional hazard models to obtain hazard ratios (HRs) with 95% CIs for incident depression and anxiety using linked hospital admissions data as the source. Hospital admissions data was obtained via record linkage to different sources including Hospital Episode Statistics (England), Scottish Morbidity Record (Scotland), and Patient Episode Database (Wales). Participants with incident depression and anxiety were identified as having the first record using International Classification of Diseases, 10th Revision (ICD‐10) codes (F32, F33 for depression; F40, F41 for anxiety). Sixth, the associations of each food group with depressive and anxiety symptoms were tested with logistic regression. Seventh, considering depression and anxiety could be precipitated by interacting factors from various stress sources (e.g., work-related, sleep quality), the model was further adjusted for sleep score, length of the working week for the primary job, and shift work involvement. Eighth, considering that depression and anxiety are frequently comorbid with other psychiatric comorbidities, logistic regression models were further adjusted for attention deficit hyperactivity disorder (ADHD) and eating disorders. Stata version 17.0 (StataCorp) was used to conduct RRR. R software (version 4.0.5) was used for the remaining statistical analyses. Two-sided* P* < 0.05 indicated statistical significance.

## Results

### Population characteristics

Seventy-five thousand four hundred sixty-six participants (mean age 55.8 years, 55.9% women) were finally included in this study and followed up for an average of 7.6 years until the PHQ-9 and GAD-7 questionnaires were completed. 2746 depressive symptoms and 2202 anxiety symptoms were recorded during this period, respectively. Table 1 shows the baseline characteristics of the study participants according to depression and anxiety status. Additional file 1: Table S2 shows the baseline characteristics of the study participants according to three dietary patterns.

**Table 1:** Baseline characteristics of participants by outcomes (*N* = 75,466)

Characteristics	Total (*N* = 75,466)	Depressive symptoms			Anxiety symptoms		
		**No** **(*N* = 71,649)**	**Yes** **(*N* = 3817)**	*P* value^*^	**No** **(*N* = 72,557)**	**Yes** **(*N* = 2909)**	*P* value^*^
Female, *n* (%)	42,192 (55.9)	39,785 (55.5)	2407 (63.1)	< 0.001	40,233 (55.5)	1959 (67.3)	< 0.001
Age (years)^a^	55.8 (7.7)	56.0 (7.7)	52.6 (7.7)	< 0.001	56.0 (7.7)	52.9 (7.7)	< 0.001
TDI^a^	− 1.7 (2.8)	− 1.7 (2.8)	− 0.9 (3.1)	< 0.001	− 1.7 (2.8)	− 1.1 (3.1)	< 0.001
Education level, *n* (%)				< 0.001			< 0.001
College qualification	49,207 (65.2)	47,088 (65.7)	2119 (55.5)		47,521 (65.5)	1686 (58.0)	
Other qualification	22,313 (29.6)	20,881 (29.1)	1432 (37.5)		21,286 (29.3)	1027 (35.3)	
None qualification	3946 (5.2)	3680 (5.1)	266 (7.0)		3750 (5.2)	196 (6.7)	
Smoking status, *n* (%)				< 0.001			< 0.001
Never	44,058 (58.4)	42,074 (58.7)	1984 (52.0)		42,506 (58.6)	1552 (53.4)	
Previous	26,536 (35.2)	25,156 (35.1)	1380 (36.2)		25,467 (35.1)	1069 (36.7)	
Current	4872 (6.5)	4419 (6.2)	453 (11.9)		4584 (6.3)	288 (9.9)	
Physical activity (IPAQ), *n* (%)				< 0.001			< 0.001
Low	12,072 (16.0)	11,238 (15.7)	834 (21.8)		11,546 (15.9)	526 (18.1)	
Moderate	28,478 (37.7)	27,128 (37.9)	1350 (35.4)		27,481 (37.9)	997 (34.3)	
High	24,793 (32.9)	23,695 (33.1)	1098 (28.8)		23,836 (32.9)	957 (32.9)	
Missing data	10,123 (13.4)	9588 (13.4)	535 (14.0)		9694 (13.4)	429 (14.7)	
Ethnicity, *n* (%)				0.002			< 0.001
White	73,403 (97.3)	69,721 (97.3)	3682 (96.5)		70,611 (97.3)	2792 (96.0)	
Others	2063 (2.7)	1928 (2.7)	135 (3.5)		1946 (2.7)	117 (4.0)	
Hypertension, *n* (%)	37,230 (49.3)	35,413 (49.4)	1817 (47.6)	0.028	35,883 (49.5)	1347 (46.3)	< 0.001
Cardiovascular disease, *n* (%)	3228 (4.3)	2990 (4.2)	238 (6.2)	< 0.001	3063 (4.2)	165 (5.7)	< 0.001
Diabetes, *n* (%)	2458 (3.3)	2241 (3.1)	217 (5.7)	< 0.001	2336 (3.2)	122 (4.2)	0.004
Nutrients intake							
Energy intake (MJ/day) ^a^	8637.1 (2041.7)	8638.9 (2026.6)	8603.0 (2308.1)	0.014	8637.8 (2033.3)	8619.1 (2241.5)	0.2
Energy density (kJ/g) ^a^	6.5 (1.4)	6.4 (1.4)	6.7 (1.7)	< 0.001	6.5 (1.4)	6.6 (1.7)	< 0.001
Saturated fatty acids (%E) ^a^	7.8 (1.9)	7.8 (1.9)	7.9 (2.0)	0.004	7.8 (1.9)	7.8 (2.0)	0.5
Free sugars (%E) ^a^	11.6 (4.9)	11.5 (4.8)	12.3 (5.9)	< 0.001	11.5 (4.8)	12.2 (5.7)	< 0.001
Fiber (g/day) ^a^	1.7 (0.5)	1.7 (0.5)	1.6 (0.5)	< 0.001	1.7 (0.5)	1.7 (0.5)	0.002
Fiber Density (g/MJ) ^a^	2.1 (0.6)	2.1 (0.6)	2.1 (0.7)	< 0.001	2.1 (0.6)	2.1 (0.6)	0.002
Main food groups (g/day)							
Chocolate and confectionery^a^	11.6 (18.7)	11.4 (18.3)	15.8 (25.0)	< 0.001	11.5 (18.4)	15.4 (24.3)	< 0.001
Butter and other normal animal-fat spreads^a^	5.2 (8.2)	5.2 (8.2)	5.1 (8.2)	0.5	5.2 (8.2)	5.0 (8.1)	0.13
High-fat cheese^a^	15.0 (16.2)	15.0 (16.1)	14.5 (17.4)	< 0.001	15.0 (16.1)	14.9 (17.9)	0.016
Added sugars and preserves^a^	8.4 (12.0)	8.4 (11.9)	8.7 (13.8)	0.001	8.4 (12.0)	8.9 (13.8)	0.2
SSBs and other sugary drinks^a^	84.6 (147.9)	83.0 (145.0)	113.9 (192.6)	< 0.001	83.7 (146.5)	107.7 (179.0)	< 0.001
Milk-based desserts^a^	24.3 (37.7)	24.2 (37.6)	25.7 (40.9)	0.8	24.3 (37.7)	25.3 (39.6)	0.5
Fresh fruit^a^	195.1 (142.9)	195.7 (142.1)	184.4 (156.3)	< 0.001	195.4 (142.4)	187.9 (154.3)	< 0.001
Vegetables^a^	188.3 (130.6)	188.7 (130.0)	180.3 (142.2)	< 0.001	188.2 (129.8)	190.2 (150.2)	0.034
Time span (years)^a^	7.4 (0.8)	7.4 (0.8)	7.4 (0.8)	0.094	7.4 (0.8)	7.4 (0.8)	0.003
Baseline depression, *n* (%)				< 0.001			< 0.001
No depression	70,271 (93.1)	67,525 (94.2)	2746 (71.9)		68,061 (93.8)	2210 (76.0)	
Depression	5195 (6.9)	4124 (5.8)	1071 (28.1)		4496 (6.2)	699 (24.0)	
Baseline anxiety, *n* (%)				< 0.001			< 0.001
No anxiety	70,070 (92.8)	67,237 (93.8)	2833 (74.2)		67,868 (93.5)	2202 (75.7)	
Anxiety	5396 (7.2)	4412 (6.2)	984 (25.8)		4689 (6.5)	707 (24.3)	

*TDI* Townsend deprivation index, *IPAQ* International Physical Activity Questionnaire, *SSBs* Sugar-sweetened beverages

^*^ANOVA or *χ*^2^ test where appropriate

^a^Mean (SD)

### Characteristics of DPs

**Fig. 2:**
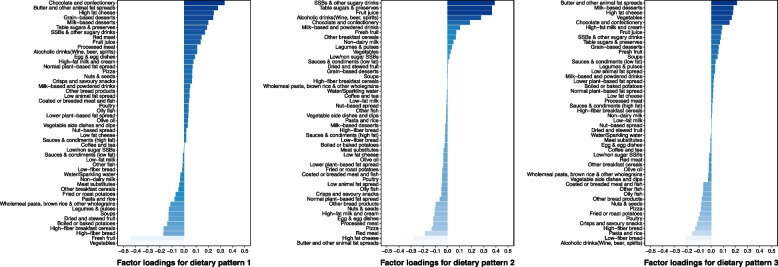
Factor loadings for food groups in dietary patterns

Four DPs were derived according to the RRR model (Additional file 1: Table S1). DP1 (44.0%), DP2 (20.0%), and DP3 (10.1%) yielded a total of 74.1% of the variation explained by energy density, free sugars, saturated fat, and fiber intakes. DP4 only explained 4.3% in all nutrient response variables. DP1 (named “high caloric diet”) was primarily loaded by high intakes of chocolate and confectionery, butter and other animal-fat spreads, and low intakes of vegetables and fresh fruit. DP2 (named “high sugar, low fat diet”) was primarily loaded by high intakes of sugar-sweetened beverages and other sugary drinks, table sugars and preserves, and low intakes of butter and other animal fat spreads, and high-fat cheese. DP3 (named “high sugar, high fat, high fiber diet”) was primarily loaded by high intakes of butter and other animal fat spreads, milk-based desserts, and low intakes of alcoholic drinks (wine, beer, spirits), and low-fiber bread (Fig. 2). In sensitivity analyses, we repeated RRR analysis with participants completing different occasions (≥ 1 to ≥ 4 times) of dietary assessments, and the results indicated the robustness of DPs derived by RRR model (Additional file 1: Fig. S2).

### Associations of DPs with depressive and anxiety symptoms

**Fig. 3:**
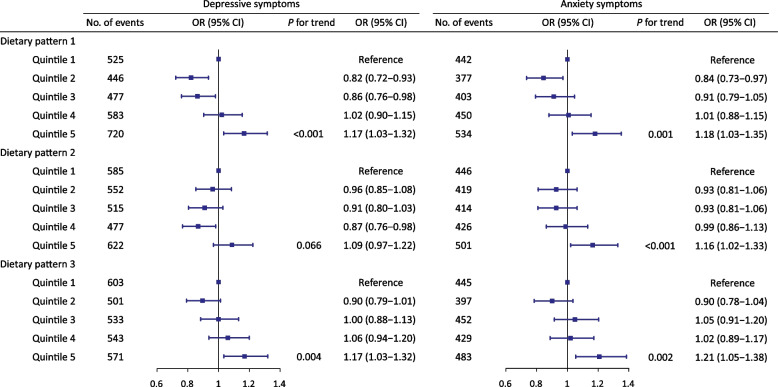
ORs (95% CIs) for depressive and anxiety symptoms by dietary patterns. All models were adjusted for age, sex, ethnicity, Townsend deprivation index, education level, smoking status, physical activity, history of hypertension, history of diabetes, and history of cardiovascular disease

**Fig. 4:**
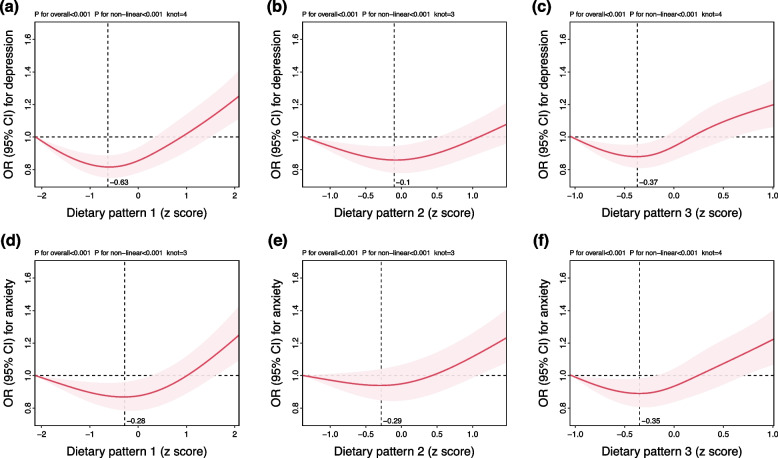
ORs (95% CIs) of continuous dietary pattern *z*-scores for the risk of depressive and anxiety symptoms. **a**–**c** Depressive symptoms. **d**–**f** Anxiety symptoms. Bold lines represent ORs, while shaded areas indicate 95% CIs. All models were adjusted for age, sex, ethnicity, Townsend deprivation index, education level, smoking status, physical activity, history of hypertension, history of diabetes, and history of cardiovascular disease

To examine relationships between DPs and depressive and anxiety symptoms, logistic regression analyses and dose–response analyses were performed. As shown in Fig. 3, we found that DP1 was associated with depressive or anxiety symptoms. Compared to the lowest quintile of DP1, the odds ratio (95% CI) of depressive symptoms was 0.82 (0.72–0.93) for Q2, 0.86 (0.76–0.98) for Q3, 1.02 (0.90–1.15) for Q4, and 1.17 (1.03–1.32) for Q5. Compared to the lowest quintile of DP1, the odds ratio (95% CI) of anxiety symptoms was 0.84 (0.73–0.97) for Q2, 0.91 (0.79–1.05) for Q3, 1.01 (0.88–1.15) for Q4, and 1.18 (1.03–1.35) for Q5. Similar association was observed between DP3 and depressive or anxiety symptoms. Compared to the lowest quintile of DP3, the odds ratio (95% CI) of anxiety symptoms was 0.90 (0.79–1.01) for Q2, 1.00 (0.88–1.13) for Q3, 1.06 (0.94–1.20) for Q4, and 1.17 (1.03–1.32) for Q5. Compared to the lowest quintile of DP3, the odds ratio (95% CI) of anxiety symptoms was 0.90 (0.78–1.04) for Q2, 1.05 (0.91–1.20) for Q3, 1.02 (0.89–1.17) for Q4, and 1.21 (1.05–1.38) for Q5. DP2 was not significantly associated with depressive symptoms (*P* for trend > 0.05), whereas a significant association was found between DP2 and anxiety symptoms (OR [95% CI] for Q5 *v.* Q1 = 1.16 [1.02 to 1.33], Q4 *v.* Q1 = 0.99 [0.86 to 1.13], Q3 *v.* Q1 = 0.93 [0.81 to 1.06], and Q2 *v.* Q1 = 0.93 [0.81 to 1.06]). After adjusting for potential confounders, restricted cubic splines showed non-linear associations between three DPs and depressive or anxiety symptoms (*P* for non-linear < 0.001) (Fig. 4). In the dose–response analysis, we found that the OR (95% CI) per standard deviation (SD) increase for DP1 with depressive symptoms was 0.82 (0.76–0.89) (DP1 *z*-score <  − 0.63) and 1.18 (1.13–1.24) (DP1 *z*-score >  − 0.63), for DP2, it was 0.93 (0.84–1.04) (DP2 z-score <  − 0.10) and 1.24 (1.15–1.33) (DP2 *z*-score >  − 0.10), and for DP3 it was 0.78 (0.65–0.94) (DP3 *z*-score <  − 0.37) and 1.27 (1.15–1.40) (DP3 *z*-score >  − 0.37). We found that the OR per SD increase for DP1 with anxiety symptoms was 0.89 (0.82–0.96) (DP1 *z*-score <  − 0.28) and 1.17 (1.10–1.24) (DP1 *z*-score >  − 0.28), for DP2, it was 0.92 (0.80–1.07) (DP2 *z*-score <  − 0.29) and 1.20 (1.11–1.29) (DP2 *z*-score >  − 0.29), and for DP3, it was 0.80 (0.65–0.99) (DP3 *z*-score <  − 0.35) and 1.29 (1.16–1.44) (DP3 *z*-score >  − 0.35) (Additional file 1: Table S3). In summary, worse dietary patterns (high sugar, fat, low fiber) were associated with higher odds of depressive and anxiety symptoms in a predominantly non-linear fashion.

### Sensitivity analyses

Several sensitivity analyses were conducted to evaluate the robustness of the primary findings. Results were consistent when using linked hospital admissions data as the source of outcomes and found non-linear significant associations between DPs and depression and anxiety (Additional file 1: Table S4 and Fig. S3). Results were similar when using the imputed data set with multiple imputations (Additional file 1: Table S5), when DPs characterized by single nutrients were taken into account (Additional file 1: Figs. S4-S6), and when food groups were taken into account (Additional file 1: Fig. S7). Results were attenuated after individual adjustment for sleep score, length of working week for the main job, and job involving shift work (Additional file 1: Figs. S8-S9); results were similar when additionally adjusted for other common psychiatric comorbidities (Additional file 1: Fig. S10).

Noteworthy, there were significant interactions between DP1 and sex and physical activity, as well as DP3 and age. The association between DP1 and depressive symptoms was more pronounced in males (OR = 1.12; 95% CI: 1.07–1.18) than females (OR = 1.03; 95% CI: 0.98–1.07). The association between DP1 and anxiety symptoms was more pronounced among individuals with physical inactive (OR = 1.08; 95% CI: 1.03–1.14) than physical active (OR = 1.01; 95% CI: 0.96–1.07). The association between DP3 and anxiety symptoms was more pronounced among older people (OR = 1.20; 95% CI: 1.02–1.40) (Additional file 1: Fig. S11).

## Discussion

Utilizing data of 75,466 participants from UK Biobank, this study investigated the associations between dietary patterns characterized by energy density, saturated fat, free sugars, and fiber and depressive and anxiety symptoms, with three main dietary patterns derived. DP1 explained the most variance in nutrient intakes (44%) and energy density was one of the most explained (65.4%). DP1 was characterized by high intakes of energy, saturated fatty acids, free sugars, and low intakes of fiber. There were non-linear associations between DP1 with depressive and anxiety symptoms. DP2 explained less of the variance in nutrient intakes (20%), in which free sugar was one of the most explained nutrients (56.1%). It was characterized by high intakes of free sugars, and low intakes of saturated fatty acids, but no significant associations with depressive and anxiety symptoms were observed. DP3 explained much less variance (10%) and saturated fatty acid was one of the most explained nutrients (23.2%), along with high intakes of saturated fatty acids, free sugars and fiber, and low intakes of energy. In the main analyses, DP3 was non-linearly associated with depressive and anxiety symptoms, with stronger associations found among people aged ≥ 60 years for anxiety symptoms. The findings were largely consistent in a series of sensitivity analyses.

Our findings highlight the intricate relationships between overall diet pattern and symptoms of depression and anxiety. The current analysis has yielded robust findings that increased consumption of free sugars and saturated fatty acids was associated with a heightened risk of symptoms related to depression and anxiety. Previous published studies have shown inconsistent associations between saturated fatty acids or free sugars and common mental disorders. In an observational study of 12,059 Spanish university graduates, no correlation between saturated fatty acids and depression was found [7]. A Whitehall II cohort study based on 5044 middle-aged British adults show that dietary patterns characterized by high sugar were not associated with depression [32]. Moreover, in a prospective analysis based on 69,954 women from the Women’s Health Initiative cohort, an inverse correlation was reported between fiber intake and the risk of depression [33]. However, we found that a moderate intake of dietary fiber was associated with a lower risk of depressive and anxiety symptoms. Conversely, inadequate or excessive dietary fiber may elevate the risk of experiencing depressive and anxiety symptoms, as evidenced by a U-shaped relationship. In observational analyses, the investigation of nutrient intake and anxiety remains limited [22].

The DP1 in this study, referred to as a “high-calorie diet,” was found to be associated with an increased risk of depressive and anxiety symptoms. In the present study, we found excessive consumption of butter and other animal-fat spreads, high-fat cheese, and low consumption of fresh fruit and vegetables in DP1 was significantly associated with depressive symptoms, which was in line with the previous study [12, 34]. DP2 was an abnormal dietary pattern with high intakes of saturated fatty acids mainly distributed at quintile 1 (9.4%) and remarkably high intakes of free sugars at quintile 5 (17.5%), which lead to null association with depressive symptoms in logistic regression analyses. DP2 can be named as “high sugar, low fat diet”. However, DP2 showed a significant ascending trend with depressive symptoms in the restricted cubic splines analyses, indicating that excessive intakes of high-sugar foods or drinks (e.g., sugar-sweetened beverages, table sugars and preserves) were associated with an increased risk of depression. Meanwhile, a significant non-linear association between DP2 and anxiety was found, which confirmed the U-shaped trend between nutrients and anxiety, and indicated that food intakes characterized by inadequate intakes of saturated fatty acids and excessive intakes of free sugars combined (e.g., high intakes of sugar-sweetened beverages and low intakes of high-fat cheese) increased the risk of anxiety, and this finding was not investigated ever before. DP3 is a hybrid dietary pattern that can be named as “high sugar, high fat, high fiber diet”. As against DP1, DP3 had a relatively low level of energy density (6.2% *v.* 8.2%) and free sugars (13.8% *v.* 15.1%) at quintile 5, indicating that people in DP3 quintile 5 consumed less energy-dense foods. Unfortunately, we did not find any useful interaction between DP3 and given risk factors, so the mechanism for DP1–DP3 difference is unclear.

Using the RRR approach, our findings of curvilinear relation between nutrients suggest to limit intakes of chocolate and confectionery, butter and other animal fat spreads, high-fat cheese, sugar-sweetened beverages, and other sugary drinks, table sugars and preserves, and milk-based desserts. The excessive consumption of these foods may trigger systemic inflammation, increase oxidative stress, and cause alterations in gut microbiota [35], thereby contributing to the risk of developing symptoms of depression and anxiety.

Several strengths should be acknowledged. A strength of this study is that we applied both self-reported scales and clinical diagnosis as the source of depression and anxiety. Besides, we did not assume linearity between dietary patterns and depression and anxiety, and we investigated links between single nutrients and depression and anxiety in the sensitivity analyses, which further supported the non-linear relationship. Furthermore, we excluded individuals with depression and anxiety at baseline to avoid reverse causation. Most importantly, we used the reduced rank regression method to obtain objective dietary scores. This approach provides a more robust and reliable analysis of the relationship between dietary patterns and mental health outcomes.

Several limitations should also be acknowledged. First, we used PHQ-9 and GAD-7 questionnaires as the source of depression and anxiety, which are subjective and have a limited accuracy. For example, as reported by a recent study, PHQ-9 (with cut-off score ≥ 10) has a sensitivity of 88% (95% CI: 80 to 90%) and specificity of 85% (95% CI: 82 to 87%) [24], which means still some of the people are under-detected. Considering this limitation, we performed sensitive analyses using clinical diagnosis as the source of depression and anxiety and yielded robust results. Second, although we have taken measures to exclude patients with pre-existing depression and anxiety at baseline by incorporating hospital admissions data and antidepressant medication status, the unique nature of depression and anxiety diagnoses still prevents us from ensuring the complete inclusion of all cases. However, the diagnoses of depression and anxiety, from linking to hospital admissions data, have been validated against detailed clinical evaluations, and exhibit good positive predictive value (75%) [36, 37]. Third, as the 24-h dietary data were provided by a small subset of participants, we could not roll out the selection bias. Forth, intakes of each food item are self-reported, and we did not carefully exclude participants with misreporting energy intakes, so this may lead to the misreporting bias. However, we repeated the main analyses with participants completing the questionnaire with 1 to 5 occasions, implying consistent results. Fifth, time-varying exposure and covariates were not considered due to the lack of repeated measurement data in the UK Biobank. Though we calculated the mean intakes of the five times of dietary questionnaires, our estimates of dietary pattern within a short follow-up period capture only the most recent segment of lifetime’s exposure. Finally, choice of how many DPs need to retain is subjective, and the selection of nutrients is narrow that could not reflect and explain the whole pathway of depressive and anxiety symptoms, so future validation is needed.

## Conclusions

The present study revealed that a dietary pattern characterized by elevated consumption of chocolate and confectionery, butter, high-fat cheese, added sugars, and milk-based desserts, coupled with reduced intake of fresh fruit and vegetables, was associated with higher risks of depressive and anxiety symptoms. Our findings highlight that moderate consumption of foods and beverages may contribute to reducing the current burden of mental disorders at the population level.

### Supplementary Information

**Additional file 1: Table S1.** Explained variation in food intake and nutrient response variables for each DP and correlation coefficient between DPs and response variables. **Table S2.** Baseline characteristics of participants in DP 1, DP 2 and DP 3. **Table S3.** Cut-off points and OR (95% CI) per SD increase in the dose-response analysis. **Table S4.** HRs (95% CIs) of depression and anxiety from linked hospital admissions data associated with each z-score increase in DPs. **Table S5.** Completing all missing covariates using multiple imputation with 10 imputations. ORs (95% CIs) for depression and anxiety by DPs. **Figure S1.** Theoretical direct acyclic graph guiding the analyses. **Figure S2.** Factor loadings for food groups in DPs with participants completing 1, 3, 4, 5 times of 24-h dietary questionnaires. **Figure S3.** HRs (95% CIs) of continuous DP z-scores for the risk of depression and anxiety from linked hospital admissions data. **Figure S4.** ORs (95% CIs) for associations between DPs characterized by single nutrients and depression and anxiety. **Figure S5.** ORs (95% CIs) of continuous single nutrients DPs z-scores for the risk of depression and anxiety. **Figure S6.** HRs (95% CIs) of continuous single nutrients DP z-scores for the risk of depression and anxiety from linked hospital admissions data. **Figure S7.** ORs (95% CIs) for depression and anxiety by each food groups. **Figure S8.** ORs (95% CIs) for depression and anxiety by DPs after further adjustment for sleep score, length of the working week for the primary job, and shift work involvement. **Figure S9.** ORs (95% CIs) of DP for the risk of depression and anxiety after further adjustment for sleep score, length of the working week for the primary job, and shift work involvement. **Figure S10.** ORs (95% CIs) for depression and anxiety by DPs after further adjustment for ADHD, and eating disorders. **Figure S11.** ORs (95% CIs) for associations between DPs and depression and anxiety modified by risk factors.

## Data Availability

The data that support the findings of this study are available from the UK Biobank project site, subject to registration and application process. Further details can be found at https://www.ukbiobank.ac.uk.

## Abbreviations

CI: Confidence interval
DP: Dietary pattern
GAD-7: General Anxiety Disorder-7
ICD: International Classification of Diseases
OR: Odds ratio
PHQ-9: Patient Health Questionnaire-9
RRR: Reduced rank regression
SD: Standard deviation
SSBs: Sugar-sweetened beverages
