# Supplementary material for: The associations of dietary patterns with depressive and anxiety symptoms: a prospective study
Source: BMC Med. 2023 Aug 15;21:307. doi: 10.1186/s12916-023-03019-x (PMC10426158; doi:10.1186/s12916-023-03019-x)
Supplement: Supplementary file 1 — Additional file 1: Table S1. Explained variation in food intake and nutrient response variables for each DP and correlation coefficient between DPs and response variables. Table S2. Baseline characteristics of participants in DP 1, DP 2 and DP 3. Table S3. Cut-off points and OR (95% CI) per SD increase in the dose-response analysis. Table S4. HRs (95% CIs) of depression and anxiety from linked hospital admissions data associated with each z-score increase in DPs. Table S5. Completing all missing covariates using multiple imputation with 10 imputations. ORs (95% CIs) for depression and anxiety by DPs. Figure S1. Theoretical direct acyclic graph guiding the analyses. Figure S2. Factor loadings for food groups in DPs with participants completing 1, 3, 4, 5 times of 24-h dietary questionnaires. Figure S3. HRs (95% CIs) of continuous DP z-scores for the risk of depression and anxiety from linked hospital admissions data. Figure S4. ORs (95% CIs) for associations between DPs characterized by single nutrients and depression and anxiety. Figure S5. ORs (95% CIs) of continuous single nutrients DPs z-scores for the risk of depression and anxiety. Figure S6. HRs (95% CIs) of continuous single nutrients DP z-scores for the risk of depression and anxiety from linked hospital admissions data. Figure S7. ORs (95% CIs) for depression and anxiety by each food groups. Figure S8. ORs (95% CIs) for depression and anxiety by DPs after further adjustment for sleep score, length of the working week for the primary job, and shift work involvement. Figure S9. ORs (95% CIs) of DP for the risk of depression and anxiety after further adjustment for sleep score, length of the working week for the primary job, and shift work involvement. Figure S10. ORs (95% CIs) for depression and anxiety by DPs after further adjustment for ADHD, and eating disorders. Figure S11. ORs (95% CIs) for associations between DPs and depression and anxiety modified by risk factors. [file 12916_2023_3019_MOESM1_ESM.docx]

**Supplementary Materials**

**Additional Table S1** Explained variation in food intake and nutrient response variables for each DP and correlation coefficient between DPs and response variables (*N*=126 257).

**Additional Table S2** Baseline characteristics of participants in dietary pattern 1, dietary pattern 2 and dietary pattern 3 (*N*=75 466).

**Additional Table S3** Cut-off points and OR (95% CI) per standard deviation (SD) increase in the dose-response analysis.

**Additional Table S4** Linked hospital admissions data as the source of outcomes. HRs (95% CIs) of depression and anxiety associated with each z-score increase in DPs.

**Additional Table S5** Completing all missing covariates using multiple imputation with 10 imputations. ORs (95% CIs) for depression and anxiety by DPs.

**Additional Figure S1** Theoretical direct acyclic graph guiding the analyses.

**Additional Figure S2** Factor loadings for food groups in dietary patterns with participants completing 1, 3, 4, 5 times of 24-h dietary questionnaires.

**Additional Figure S3** HRs (95% CIs) of continuous dietary pattern z-scores for the risk of depression and anxiety using linked hospital admissions data as the source of outcomes.

**Additional Figure S4** ORs (95% CIs) for associations between DPs characterized by single nutrients and depression and anxiety.

**Additional Figure S5** ORs (95% CIs) of continuous single nutrients DPs z-scores for the risk of depression and anxiety.

**Additional Figure S6** HRs (95% CIs) of continuous single nutrients DPs z-scores for the risk of depression and anxiety using linked hospital admissions data as the source of outcomes.

**Additional Figure S7** ORs (95% CIs) for depression and anxiety by each food groups.

**Additional Figure S8** ORs (95% CIs) for depression and anxiety by dietary patterns after further adjustment for sleep score, length of the working week for the primary job, and shift work involvement.

**Additional Figure S9** ORs (95% CIs) of continuous dietary pattern z-scores for the risk of depression and anxiety after further adjustment for sleep score, length of the working week for the primary job, and shift work involvement.

**Additional Figure S10** ORs (95% CIs) for depression and anxiety by dietary patterns after further adjustment for attention deficit hyperactivity disorder, and eating disorders.

**Additional Figure S11** ORs (95% CIs) for associations between DPs and depression and anxiety modified by risk factors.

**Additional Table S1** Explained variation in food intake and nutrient response variables for each DP and correlation coefficient between DPs and response variables (*N*=126 257).

| Dietary pattern (DP) | Explained variation (%) | | | | | | Correlation coefficient | | | |
| --- | --- | --- | --- | --- | --- | --- | --- | --- | --- | --- |
|  | Food intakes | Nutrient response  variables | Energy  Density  (kJ/g) | Saturated  fat acids (%E) | Free sugars (%E) | Fiber density(g/MJ) | Energy  Density  (kJ/g) | Saturated  fat acids (%E) | Free sugars (%E) | Fiber density(g/MJ) |
| DP1 | 4.08 | 44.0 | 65.4 | 25.9 | 22.0 | 62.8 | 0.61 | 0.38 | 0.35 | -0.60 |
| DP2 | 2.06 | 20.0 | 1.8 | 21.9 | 56.1 | 0 | -0.15 | -0.52 | 0.84 | 0 |
| DP3 | 2.55 | 10.1 | 4.9 | 23.2 | 6.7 | 5.6 | -0.35 | 0.76 | 0.41 | 0.37 |
| DP4 | 2.36 | 4.3 | 8.3 | 0.1 | 0.1 | 8.7 | 0.70 | -0.07 | 0.08 | 0.71 |
| Note: %E, proportion of total energy intake. | | | | | | | | | | |

**Additional Table S2** Baseline characteristics of participants in dietary pattern 1, dietary pattern 2 and dietary pattern 3 (*N*=75 466).

| Characteristics | Total  (*N* = 75 466) | Dietary pattern 1 | | | |  | Dietary pattern 2 | | | |  | Dietary pattern 3 | | | |
| --- | --- | --- | --- | --- | --- | --- | --- | --- | --- | --- | --- | --- | --- | --- | --- |
|  |  | Quintile 1   (*N* = 15 094) | Quintile 3  (*N* = 15 093) | Quintile 5   (*N* = 15 093) | *P* value^*^ |  | Quintile 1  (*N* = 15 094) | Quintile 3  (*N* = 15 093) | Quintile 5   (*N* = 15 093) | *P* value^*^ |  | Quintile 1   (*N* = 15 094) | Quintile 3   (*N* = 15 093) | Quintile 5   (*N* = 15 093) | *P*  value^*^ |
| Female, n (%) | 42 192 (55.9) | 10 338 (68.5) | 8 793 (58.3) | 5 633 (37.3) | <0.001 |  | 8 460 (56.0) | 9 268 (61.4) | 6 564 (43.5) | <0.001 |  | 5 234 (34.7) | 9 398 (62.3) | 9 468 (62.7) | <0.001 |
| Age (years)^†^ | 55.8 (7.7) | 57.0 (7.3) | 56.1 (7.6) | 54.2 (8.0) | <0.001 |  | 55.7 (7.7) | 56.1 (7.6) | 55.4 (7.9) | <0.001 |  | 54.1 (7.8) | 56.1 (7.6) | 57.0 (7.5) | <0.001 |
| TDI^†^ | -1.7 (2.8) | -1.7 (2.8) | -1.8 (2.8) | -1.5 (2.9) | <0.001 |  | -1.5 (2.8) | -1.8 (2.8) | -1.6 (2.9) | <0.001 |  | -1.6 (2.9) | -1.7 (2.8) | -1.7 (2.8) | <0.001 |
| Education, n (%) |  |  |  |  | <0.001 |  |  |  |  | <0.001 |  |  |  |  | <0.001 |
| College qualification | 49 207 (65.2) | 10 237 (67.8) | 9 980 (66.1) | 9 168 (60.7) |  |  | 10 045 (66.5) | 9 785 (64.8) | 9 448 (62.6) |  |  | 9 204 (61.0) | 9 897 (65.6) | 10 275 (68.1) |  |
| Other qualification | 22 313 (29.6) | 4 075 (27.0) | 4 379 (29.0) | 5 067 (33.6) |  |  | 4 300 (28.5) | 4 513 (29.9) | 4 793 (31.8) |  |  | 5 067 (33.6) | 4 431 (29.4) | 4 024 (26.7) |  |
| None qualification | 3 946 (5.2) | 782 (5.2) | 734 (4.9) | 858 (5.7) |  |  | 749 (5.0) | 795 (5.3) | 852 (5.6) |  |  | 823 (5.5) | 765 (5.1) | 794 (5.3) |  |
| Smoking status, n (%) |  |  |  |  | <0.001 |  |  |  |  | <0.001 |  |  |  |  | <0.001 |
| Never | 44 058 (58.4) | 9 040 (59.9) | 9 002 (59.6) | 8 291 (54.9) |  |  | 8 544 (56.6) | 8 943 (59.3) | 8 707 (57.7) |  |  | 7 814 (51.8) | 8 982 (59.5) | 9 404 (62.3) |  |
| Previous | 26 536 (35.2) | 5 458 (36.2) | 5 246 (34.8) | 5 095 (33.8) |  |  | 5 456 (36.1) | 5 348 (35.4) | 5 156 (34.2) |  |  | 5 994 (39.7) | 5 245 (34.8) | 4 773 (31.6) |  |
| Current | 4 872 (6.5) | 596 (3.9) | 845 (5.6) | 1 707 (11.3) |  |  | 1 094 (7.2) | 802 (5.3) | 1 230 (8.1) |  |  | 1 286 (8.5) | 866 (5.7) | 916 (6.1) |  |
| Physical activity (IPAQ), n (%) |  |  |  |  | <0.001 |  |  |  |  | <0.001 |  |  |  |  | <0.001 |
| Low | 12 072 (16.0) | 1 724 (11.4) | 2 435 (16.1) | 3 134 (20.8) |  |  | 2 623 (17.4) | 2 345 (15.5) | 2 330 (15.4) |  |  | 2 761 (18.3) | 2 353 (15.6) | 2 180 (14.4) |  |
| Moderate | 28 478 (37.7) | 5 496 (36.4) | 5 843 (38.7) | 5 585 (37.0) |  |  | 5 668 (37.6) | 5 728 (38.0) | 5 560 (36.8) |  |  | 5 575 (36.9) | 5 783 (38.3) | 5 546 (36.7) |  |
| High | 24 793 (32.9) | 5 913 (39.2) | 4 795 (31.8) | 4 471 (29.6) |  |  | 4 808 (31.9) | 4 913 (32.6) | 5 305 (35.1) |  |  | 4 907 (32.5) | 4 828 (32.0) | 5 309 (35.2) |  |
| Missing data | 10 123 (13.4) | 1 961 (13.0) | 2 020 (13.4) | 1 903 (12.6) |  |  | 1 995 (13.2) | 2 107 (14.0) | 1 898 (12.6) |  |  | 1 851 (12.3) | 2 129 (14.1) | 2 058 (13.6) |  |
| Ethnicity, n (%) |  |  |  |  | 0.005 |  |  |  |  | <0.001 |  |  |  |  | <0.001 |
| White | 73 403 (97.3) | 14 616 (96.8) | 14 679 (97.3) | 14 711 (97.5) |  |  | 14 774 (97.9) | 14 715 (97.5) | 14 526 (96.2) |  |  | 14 528 (96.3) | 14 702 (97.4) | 14 776 (97.9) |  |
| Others | 2 063 (2.7) | 478 (3.2) | 414 (2.7) | 382 (2.5) |  |  | 320 (2.1) | 378 (2.5) | 567 (3.8) |  |  | 566 (3.7) | 391 (2.6) | 317 (2.1) |  |
| Hypertension, n (%) | 37 230 (49.3) | 7 420 (49.2) | 7 411 (49.1) | 7 569 (50.1) | 0.12 |  | 7 407 (49.1) | 7 334 (48.6) | 7 761 (51.4) | <0.001 |  | 8 066 (53.4) | 7 209 (47.8) | 7 155 (47.4) | <0.001 |
| Cardiovascular disease, n (%) | 3 228 (4.3) | 679 (4.5) | 652 (4.3) | 634 (4.2) | 0.6 |  | 562 (3.7) | 647 (4.3) | 754 (5.0) | <0.001 |  | 754 (5.0) | 611 (4.0) | 603 (4.0) | <0.001 |
| Diabetes, n (%) | 2 458 (3.3) | 572 (3.8) | 496 (3.3) | 450 (3.0) | <0.001 |  | 831 (5.5) | 444 (2.9) | 276 (1.8) | <0.001 |  | 677 (4.5) | 482 (3.2) | 364 (2.4) | <0.001 |
| Nutrients intake |  |  |  |  |  |  |  |  |  |  |  |  |  |  |  |
| Energy intake (MJ/day) ^†^ | 8 637.1  (2 041.7) | 8 099.4  (1 892.3) | 8 349.9  (1 857.2) | 9 908.3  (2 152.7) | <0.001 |  | 9 192.0  (2 105.3) | 8 253.4  (1 942.2) | 9 006.3  (2 083.8) | <0.001 |  | 9 124.0  (2 102.1) | 8 198.0  (1 914.8) | 9 194.6  (2 062.3) | <0.001 |
| Energy density (kJ/g) ^†^ | 6.5 (1.4) | 4.9 (0.7) | 6.4 (0.8) | 8.2 (1.3) | <0.001 |  | 7.0 (1.4) | 6.3 (1.4) | 6.4 (1.5) | <0.001 |  | 7.1 (1.4) | 6.3 (1.4) | 6.2 (1.5) | <0.001 |
| Saturated fatty acids (%E) ^†^ | 7.8 (1.9) | 6.4 (1.5) | 7.8 (1.6) | 9.2 (1.9) | <0.001 |  | 9.4 (1.8) | 7.6 (1.7) | 6.8 (1.7) | <0.001 |  | 6.6 (1.5) | 7.7 (1.6) | 9.2 (2.0) | <0.001 |
| Free sugars (%E) ^†^ | 11.6 (4.9) | 8.7 (3.7) | 11.3 (4.1) | 15.1 (5.6) | <0.001 |  | 7.5 (2.9) | 10.7 (3.1) | 17.5 (4.6) | <0.001 |  | 10.1 (4.3) | 11.3 (4.5) | 13.8 (5.5) | <0.001 |
| Fiber (g/day) ^†^ | 1.7 (0.5) | 2.3 (0.4) | 1.6 (0.3) | 1.2 (0.3) | <0.001 |  | 1.6 (0.4) | 1.8 (0.5) | 1.6 (0.5) | <0.001 |  | 1.5 (0.4) | 1.7 (0.5) | 1.8 (0.5) | <0.001 |
| Fiber Density (g/MJ) ^†^ | 2.1 (0.6) | 2.8 (0.5) | 2.0 (0.3) | 1.5 (0.3) | <0.001 |  | 2.0 (0.5) | 2.2 (0.6) | 2.0 (0.6) | <0.001 |  | 1.8 (0.5) | 2.2 (0.6) | 2.2 (0.6) | <0.001 |
| Main food groups (g/day) |  |  |  |  |  |  |  |  |  |  |  |  |  |  |  |
| Chocolate and confectionery^†^ | 11.6 (18.7) | 5.4 (10.3) | 9.0 (13.4) | 24.0 (29.0) | <0.001 |  | 8.1 (13.4) | 10.5 (16.4) | 18.0 (26.4) | <0.001 |  | 7.7 (13.5) | 10.1 (15.3) | 19.4 (27.0) | <0.001 |
| Butter and other normal animal-fat spreads^†^ | 5.2 (8.2) | 2.0 (4.6) | 4.3 (6.7) | 10.1 (11.4) | <0.001 |  | 10.8 (11.5) | 3.8 (6.3) | 2.8 (5.5) | <0.001 |  | 3.1 (6.0) | 4.3 (7.0) | 9.2 (10.9) | <0.001 |
| High-fat cheese^†^ | 15.0 (16.2) | 10.6 (13.1) | 14.2 (14.8) | 21.0 (20.3) | <0.001 |  | 25.6 (21.0) | 13.0 (13.6) | 9.3 (11.9) | <0.001 |  | 10.0 (12.6) | 13.4 (13.9) | 23.5 (20.6) | <0.001 |
| Added sugars and preserves^†^ | 8.4 (12.0) | 5.1 (7.6) | 7.2 (9.4) | 14.5 (18.0) | <0.001 |  | 4.9 (7.4) | 6.8 (8.9) | 15.7 (18.3) | <0.001 |  | 6.5 (10.7) | 7.8 (11.0) | 11.8 (15.0) | <0.001 |
| SSBs and other sugary drinks^†^ | 84.6 (147.9) | 45.9 (96.1) | 73.3 (123.4) | 151.1 (211.9) | <0.001 |  | 31.8 (69.6) | 58.9 (96.0) | 201.4 (234.1) | <0.001 |  | 69.7 (127.4) | 79.8 (140.4) | 113.8 (184.4) | <0.001 |
| Milk-based desserts^†^ | 24.3 (37.7) | 14.6 (28.4) | 23.5 (35.2) | 36.0 (47.7) | <0.001 |  | 24.6 (38.9) | 24.2 (37.7) | 24.8 (38.6) | >0.9 |  | 11.0 (22.8) | 20.4 (30.6) | 46.0 (53.1) | <0.001 |
| Fresh fruit^†^ | 195.1 (142.9) | 335.0 (165.6) | 175.9 (105.2) | 101.6 (90.0) | <0.001 |  | 171.0 (132.7) | 200.9 (140.3) | 206.2 (156.1) | <0.001 |  | 150.6 (122.7) | 196.1 (135.2) | 236.8 (167.0) | <0.001 |
| Vegetables^†^ | 188.3 (130.6) | 313.7 (162.2) | 168.4 (93.0) | 110.5 (80.9) | <0.001 |  | 189.8 (128.2) | 190.7 (128.5) | 182.2 (138.6) | <0.001 |  | 134.0 (97.8) | 182.3 (112.8) | 252.2 (165.4) | <0.001 |
| Time span (years)^†^ | 7.4 (0.8) | 7.4 (0.8) | 7.4 (0.8) | 7.4 (0.8) | 0.3 |  | 7.4 (0.8) | 7.4 (0.8) | 7.4 (0.8) | 0.068 |  | 7.4 (0.8) | 7.4 (0.8) | 7.4 (0.8) | <0.001 |
| Baseline depression, n (%) |  |  |  |  | <0.001 |  |  |  |  | <0.001 |  |  |  |  | <0.001 |
| No depression | 70 271 (93.1) | 14 033 (93.0) | 14 096 (93.4) | 13 941 (92.4) |  |  | 14 132 (93.6) | 14 109 (93.5) | 13 868 (91.9) |  |  | 14 198 (94.1) | 14 040 (93.0) | 13 880 (92.0) |  |
| Depression | 5 195 (6.9) | 1 061 (7.0) | 997 (6.6) | 1 152 (7.6) |  |  | 962 (6.4) | 984 (6.5) | 1 225 (8.1) |  |  | 896 (5.9) | 1 053 (7.0) | 1 213 (8.0) |  |
| Baseline anxiety, n (%) |  |  |  |  | 0.09 |  |  |  |  | <0.001 |  |  |  |  | <0.001 |
| No anxiety | 70 070 (92.8) | 13 951 (92.4) | 14 050 (93.1) | 13 984 (92.7) |  |  | 14 111 (93.5) | 14 022 (92.9) | 13 846 (91.7) |  |  | 14 197 (94.1) | 14 006 (92.8) | 13 847 (91.7) |  |
| Anxiety | 5 396 (7.2) | 1 143 (7.6) | 1 043 (6.9) | 1 109 (7.3) |  |  | 983 (6.5) | 1 071 (7.1) | 1 247 (8.3) |  |  | 897 (5.9) | 1 087 (7.2) | 1 246 (8.3) |  |
| TDI=Townsend deprivation index; IPAQ=International Physical Activity Questionnaire; SSBs=Sugar-sweetened beverages.  ^*^ANOVA or χ2 test where appropriate; ^†^Mean (SD). | | | | | | | | | | | | | | | |

**Additional Table S3** Cut-off points and OR (95% CI) per standard deviation (SD) increase in the dose-response analysis.

| Dietary pattern (DP) | Cut-off point | OR (95% CI) per 1-SD increment^*^ | |
| --- | --- | --- | --- |
|  |  | < Cut-off point | > Cut-off point |
| **Depressive symptoms** | | | |
| DP1 | -0.63 | 0.82 (0.76-0.89) | 1.18 (1.13-1.24) |
| DP2 | -0.10 | 0.93 (0.84-1.04) | 1.24 (1.15-1.33) |
| DP3 | -0.37 | 0.78 (0.65-0.94) | 1.27 (1.15-1.40) |
| **Anxiety symptoms** | | | |
| DP1 | -0.28 | 0.89 (0.82-0.96) | 1.17 (1.10-1.24) |
| DP2 | -0.29 | 0.92 (0.80-1.07) | 1.20 (1.11-1.29) |
| DP3 | -0.35 | 0.80 (0.65-0.99) | 1.29 (1.16-1.44) |
| ^*^All models were adjusted for age, sex, ethnicity, Townsend deprivation index, education level, smoking status, physical activity, history of hypertension, history of diabetes, and history of cardiovascular disease. | | | |

|  | **Depression (*N*=111 161)**^*^ | | |  | **Anxiety (*N*=111 564)**^*^ | | |
| --- | --- | --- | --- | --- | --- | --- | --- |
|  | **Cases** | **Hazard ratio**  **(95% CI)** | ***P***  **for trend** |  | **Cases** | **Hazard ratio**  **(95% CI)** | ***P***  **for trend** |
| **DP1** | 3 929 | 1.06 (1.04,1.09) |  |  | 3 611 | 1.04 (1.01-1.07) |  |
| Quintile 1 | 763 | Reference |  |  | 759 | Reference |  |
| Quintile 2 | 752 | 1.00 (0.90,1.10) |  |  | 713 | 0.96 (0.87-1.06) |  |
| Quintile 3 | 732 | 0.99 (0.89,1.10) |  |  | 670 | 0.93 (0.84-1.04) |  |
| Quintile 4 | 758 | 1.03 (0.93,1.14) |  |  | 699 | 1.00 (0.90-1.11) |  |
| Quintile 5 | 924 | 1.31 (1.18,1.44) | <0.001 |  | 770 | 1.18 (1.07-1.31) | 0.003 |
| **DP2** | 3 929 | 1.11 (1.08,1.15) |  |  | 3 611 | 1.11 (1.07-1.15) |  |
| Quintile 1 | 800 | Reference |  |  | 699 | Reference |  |
| Quintile 2 | 732 | 0.92 (0.83,1.02) |  |  | 718 | 1.02 (0.92-1.13) |  |
| Quintile 3 | 727 | 0.93 (0.84,1.03) |  |  | 719 | 1.03 (0.93-1.15) |  |
| Quintile 4 | 761 | 1.02 (0.92,1.13) |  |  | 667 | 1.00 (0.90-1.11) |  |
| Quintile 5 | 909 | 1.27 (1.16,1.40) | <0.001 |  | 808 | 1.29 (1.16-1.43) | <0.001 |
| **DP3** | 3 929 | 1.15 (1.09,1.21) |  |  | 3 611 | 1.12 (1.06-1.18) |  |
| Quintile 1 | 685 | Reference |  |  | 606 | Reference |  |
| Quintile 2 | 755 | 1.05 (0.95,1.17) |  |  | 690 | 1.03 (0.92-1.15) |  |
| Quintile 3 | 792 | 1.09 (0.98,1.21) |  |  | 703 | 1.01 (0.91-1.13) |  |
| Quintile 4 | 803 | 1.11 (1.00,1.23) |  |  | 794 | 1.14 (1.02-1.27) |  |
| Quintile 5 | 894 | 1.26 (1.13,1.39) | <0.001 |  | 818 | 1.19 (1.06-1.32) | <0.001 |
| ^*^All models were adjusted for age, sex, ethnicity, Townsend deprivation index, education level, smoking status, physical activity, history of hypertension, history of diabetes, and history of cardiovascular disease. | | | | | | | |

**Additional Table S4** Linked hospital admissions data as the source of outcomes. HRs (95% CIs) of depression and anxiety associated with each z-score increase in DPs.

**Additional Table S5** Completing all missing covariates using multiple imputation with 10

|  | **Depression (*N*=73 328)**^*^ | | |  | **Anxiety (*N*=73 123)**^*^ | | |
| --- | --- | --- | --- | --- | --- | --- | --- |
|  | **Cases** | **Odds ratio**  **(95% CI)** | ***P***  **for trend** |  | **Cases** | **Odds ratio**  **(95% CI)** | ***P***  **for trend** |
| **DP1** |  |  |  |  |  |  |  |
| Quintile 1 | 550 | Reference |  |  | 459 | Reference |  |
| Quintile 2 | 469 | 0.83 (0.73-0.94) |  |  | 393 | 0.85 (0.74-0.98) |  |
| Quintile 3 | 492 | 0.85 (0.75-0.96) |  |  | 416 | 0.90 (0.79-1.04) |  |
| Quintile 4 | 607 | 1.01 (0.90-1.14) |  |  | 472 | 1.02 (0.89-1.16) |  |
| Quintile 5 | 754 | 1.16 (1.03-1.31) | <0.001 |  | 566 | 1.20 (1.05-1.37) | <0.001 |
| **DP2** |  |  |  |  |  |  |  |
| Quintile 1 | 617 | Reference |  |  | 465 | Reference |  |
| Quintile 2 | 569 | 0.94 (0.84-1.06) |  |  | 434 | 0.92 (0.81-1.05) |  |
| Quintile 3 | 541 | 0.91 (0.80-1.02) |  |  | 440 | 0.95 (0.83-1.08) |  |
| Quintile 4 | 495 | 0.85 (0.76-0.96) |  |  | 444 | 0.99 (0.87-1.13) |  |
| Quintile 5 | 650 | 1.07 (0.96-1.20) | 0.084 |  | 523 | 1.16 (1.02-1.32) | <0.001 |
| **DP3** |  |  |  |  |  |  |  |
| Quintile 1 | 627 | Reference |  |  | 465 | Reference |  |
| Quintile 2 | 529 | 0.91 (0.81-1.03) |  |  | 420 | 0.92 (0.80-1.05) |  |
| Quintile 3 | 558 | 1.01 (0.90-1.14) |  |  | 471 | 1.05 (0.92-1.20) |  |
| Quintile 4 | 561 | 1.06 (0.94-1.19) |  |  | 437 | 1.00 (0.87-1.15) |  |
| Quintile 5 | 597 | 1.18 (1.05-1.33) | 0.003 |  | 513 | 1.24 (1.08-1.41) | 0.001 |
| ^*^All models were adjusted for age, sex, ethnicity, Townsend deprivation index, education level, smoking status, physical activity, history of hypertension, history of diabetes, and history of cardiovascular disease. | | | | | | | |

imputations. ORs (95% CIs) for depression and anxiety by DPs.


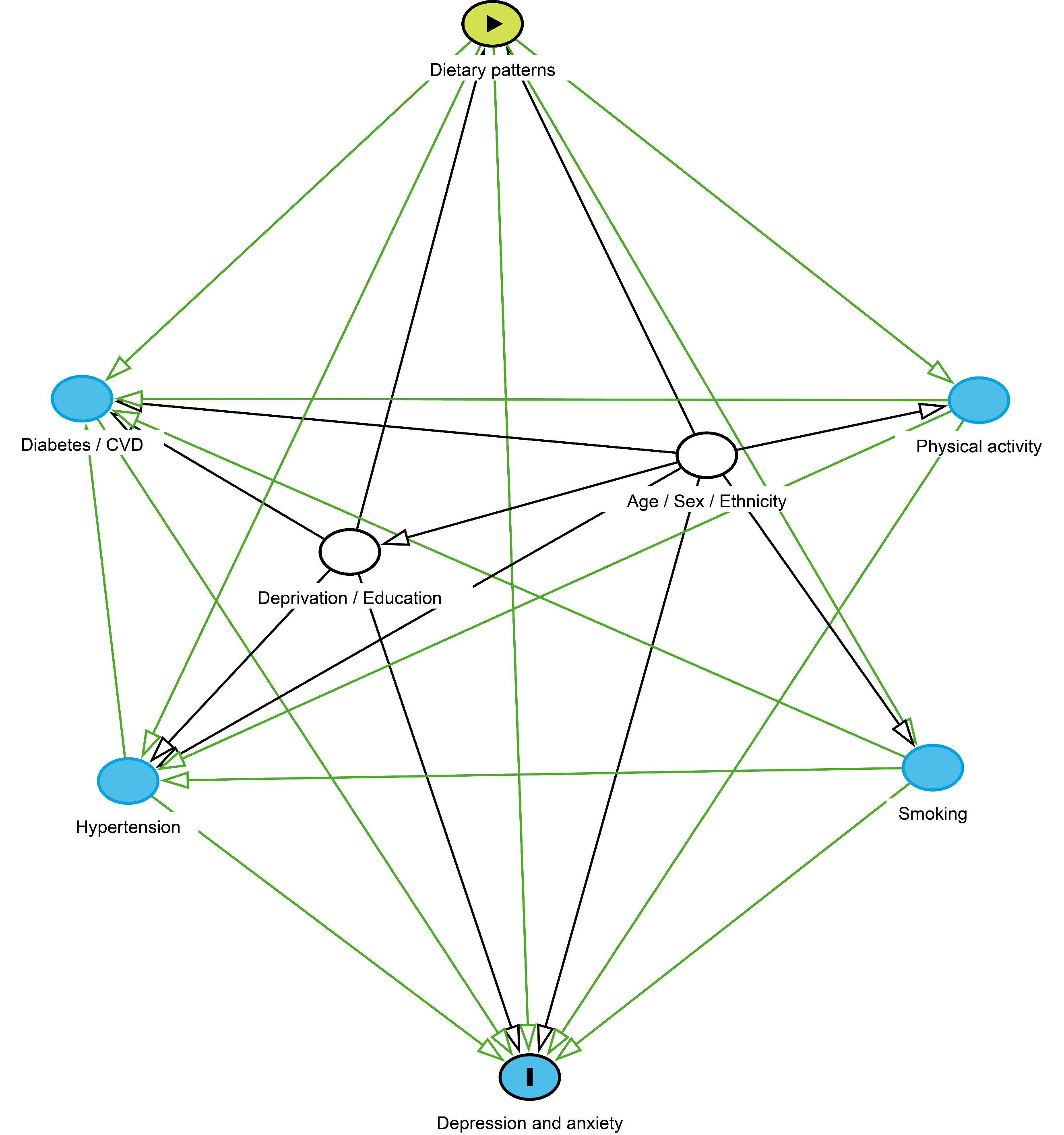


**Additional Figure S1** Theoretical direct acyclic graph guiding the analyses.


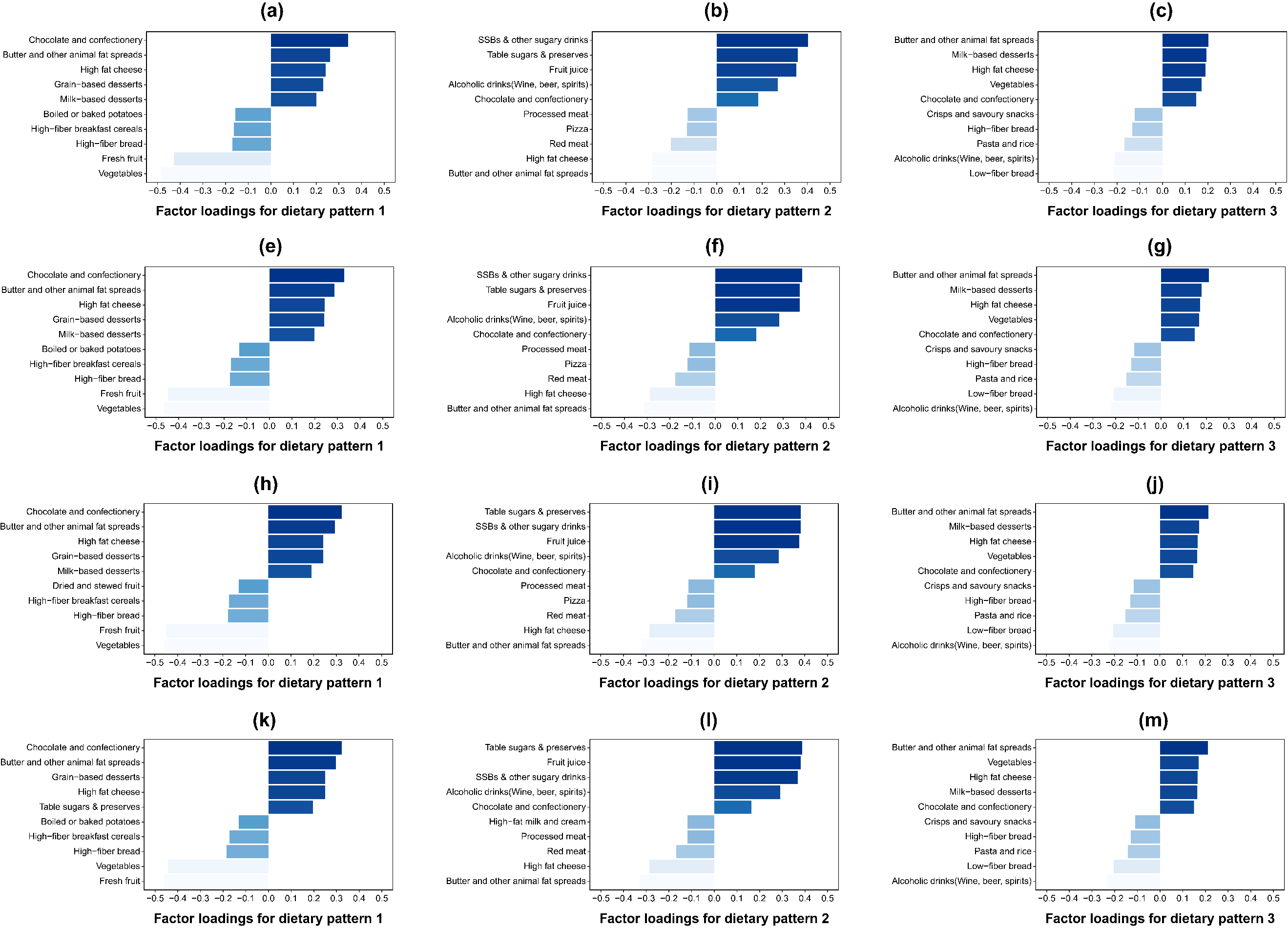
**Additional Figure S2** Factor loadings for food groups in dietary patterns with participants completing 1, 3, 4, 5 times of 24-h dietary questionnaires.

a-c: 1-times, d-f: 3-times, h-j: 4-times, k-m: 5-times.

**
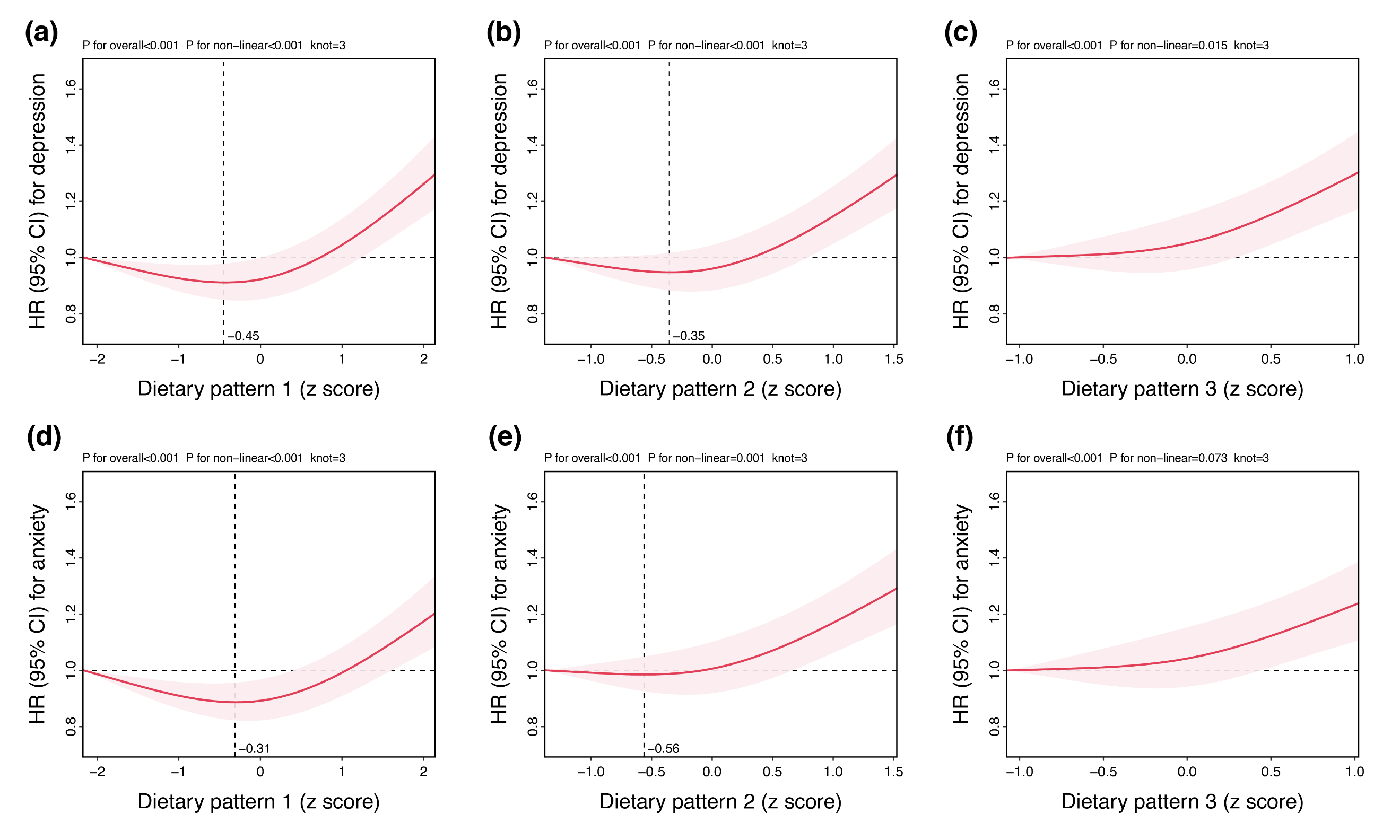
**

**Additional Figure S3** HRs (95% CIs) of continuous dietary pattern z-scores for the risk of depression and anxiety using linked hospital admissions data as the source of outcomes.

a-c: depression, d-f: anxiety.

Bold lines represent HRs, while shaded areas indicate 95% CIs. All models were adjusted for age, sex, ethnicity, Townsend deprivation index, education level, smoking status, physical activity, history of hypertension, history of diabetes, and history of cardiovascular disease.

**
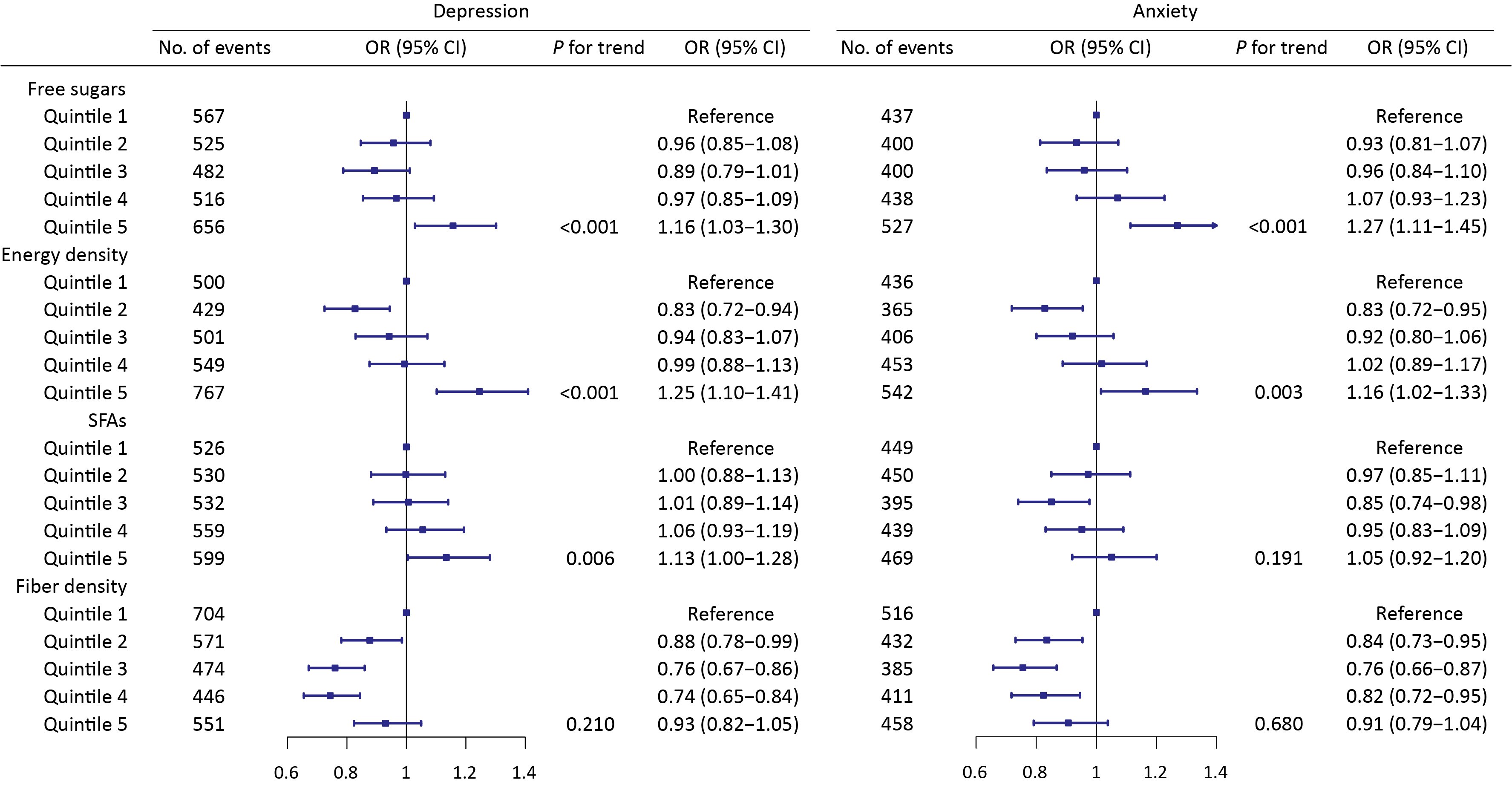
**

**Additional Figure S4** ORs (95% CIs) for associations between DPs characterized by single nutrients and depression and anxiety.

All models were adjusted for age, sex, ethnicity, Townsend deprivation index, education level, smoking status, physical activity, history of hypertension, history of diabetes, and history of cardiovascular disease.

**
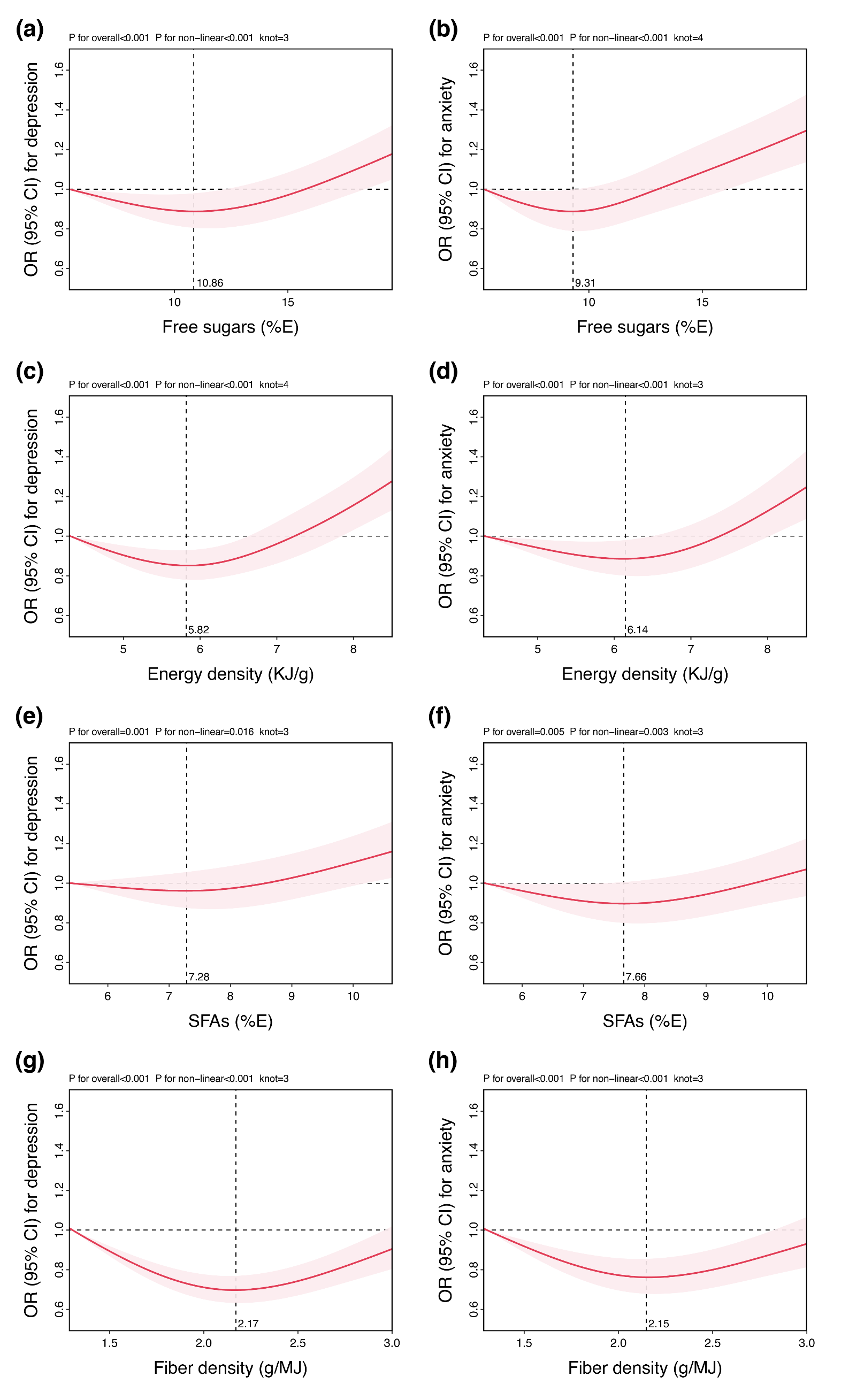
**

**Additional Figure S5** ORs (95% CIs) of continuous single nutrients DPs z-scores for the risk of depression and anxiety.

a, c, e, g: depression, b, d, f, h: anxiety.

Bold lines represent ORs, while shaded areas indicate 95% CIs. All models were adjusted for age, sex, ethnicity, Townsend deprivation index, education level, smoking status, physical activity, history of hypertension, history of diabetes, and history of cardiovascular disease.

**
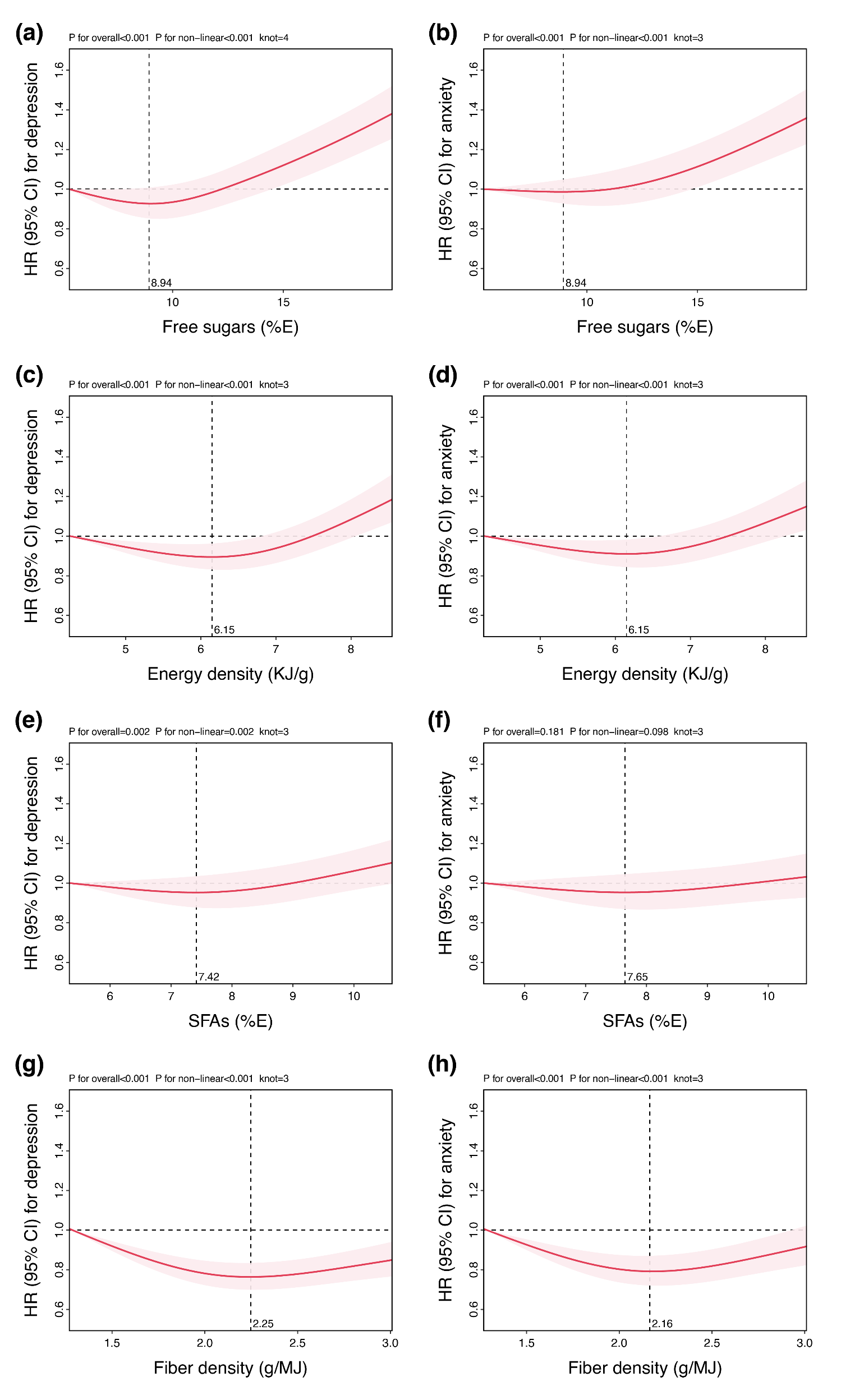
**

**Additional Figure S6** HRs (95% CIs) of continuous single nutrients DPs z-scores for the risk of depression and anxiety using linked hospital admissions data as the source of outcomes.

a, c, e, g: depression, b, d, f, h: anxiety.

Bold lines represent ORs, while shaded areas indicate 95% CIs. All models were adjusted for age, sex, ethnicity, Townsend deprivation index, education level, smoking status, physical activity, history of hypertension, history of diabetes, and history of cardiovascular disease.


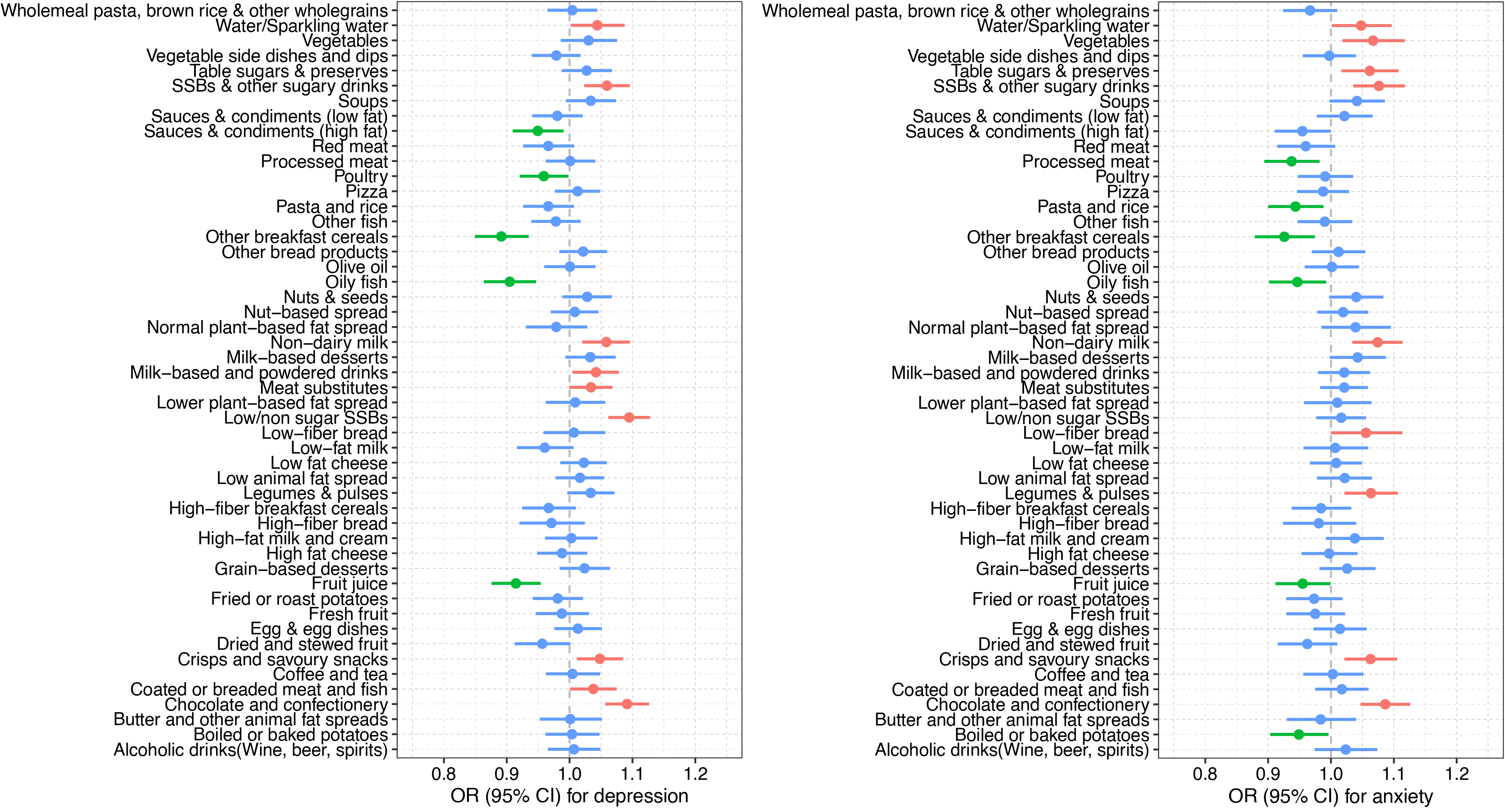


**Additional Figure S7** ORs (95% CIs) for depression and anxiety by each food groups.

Red lines indicate significant positive associations, blue lines indicate null associations, while green lines indicate significant negative associations. All models were adjusted for age, sex, ethnicity, Townsend deprivation index, education level, smoking status, physical activity, history of hypertension, history of diabetes, and history of cardiovascular disease.


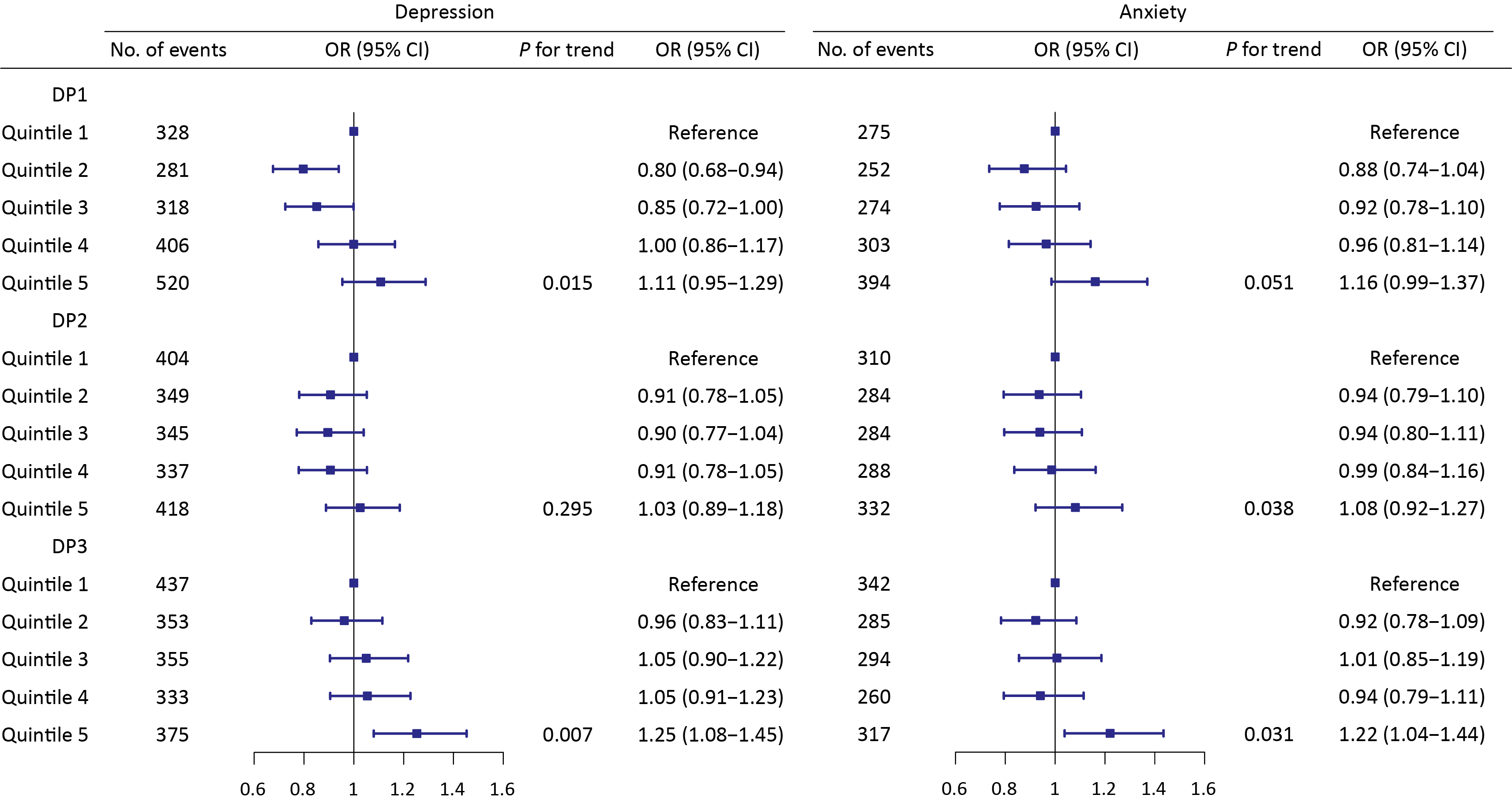


**Additional Figure S8** ORs (95% CIs) for depression and anxiety by dietary patterns after further adjustment for sleep score, length of the working week for the primary job, and shift work involvement.

All models were adjusted for age, sex, ethnicity, Townsend deprivation index, education level, smoking status, physical activity, history of hypertension, history of diabetes, history of cardiovascular disease, sleep score, length of the working week for the primary job, and shift work involvement. Sleep score was defined as: early chronotype (‘morning’ or ‘morning than evening’); sleep 7–8 h per day; reported never/rarely or sometimes insomnia symptoms; no self-reported snoring; no excessive daytime sleepiness (‘never/rarely’ or ‘sometimes’); easy getting up in morning (‘fairly easy’ or ‘very easy’); and no excessive nap during day (‘never/rarely’ or ‘sometimes’).


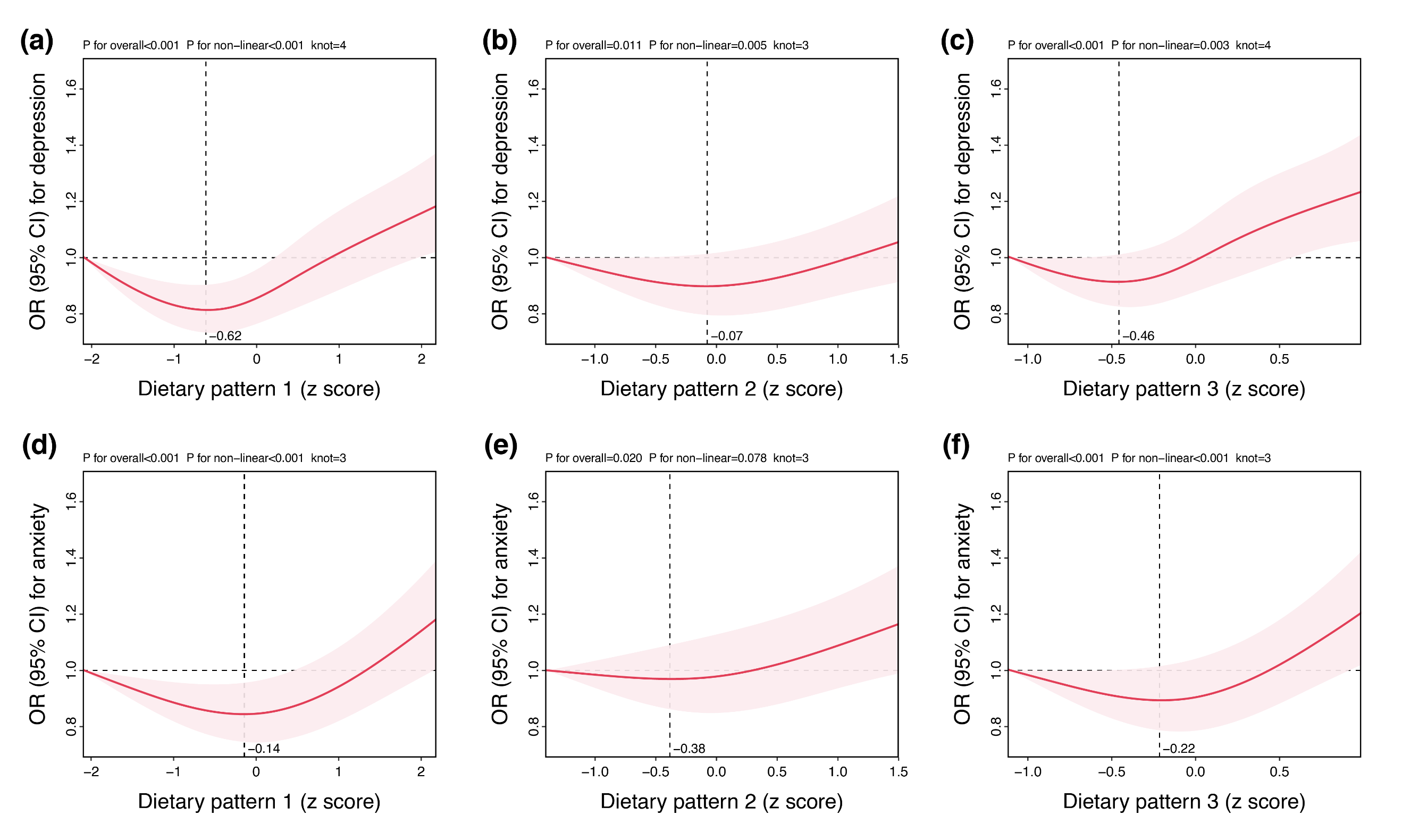


**Additional Figure S9** ORs (95% CIs) of continuous dietary pattern z-scores for the risk of depression and anxiety after further adjustment for sleep score, length of the working week for the primary job, and shift work involvement.

a-c: depression, d-f: anxiety.

Bold lines represent ORs, while shaded areas indicate 95% CIs. All models were adjusted for age, sex, ethnicity, Townsend deprivation index, education level, smoking status, physical activity, history of hypertension, history of diabetes, history of cardiovascular disease, sleep score, length of the working week for the primary job, and shift work involvement.


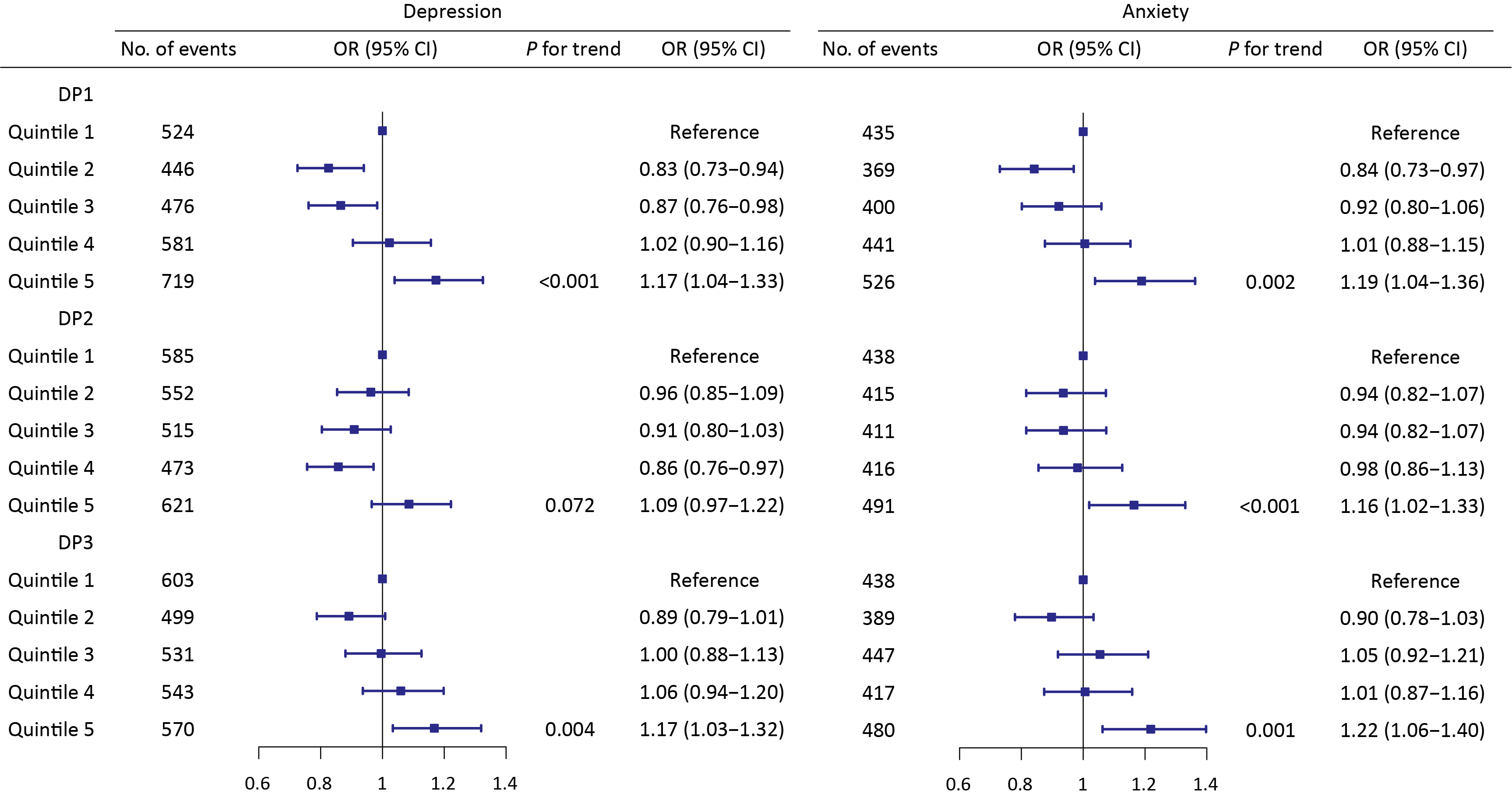


**Additional Figure S10** ORs (95% CIs) for depression and anxiety by dietary patterns after further adjustment for attention deficit hyperactivity disorder, and eating disorders.

All models were adjusted for age, sex, ethnicity, Townsend deprivation index, education level, smoking status, physical activity, history of hypertension, history of diabetes, history of cardiovascular disease, attention deficit hyperactivity disorder, eating disorders. ADHD cases were diagnosed according to the ICD-10 codes (F90.0, F90.1, F98.8) identified using hospital admissions data and medication prescribed specific for ADHD symptoms; while cases of eating disorders were diagnosed according to the ICD-10 codes (F50) identified using hospital admissions data.

**
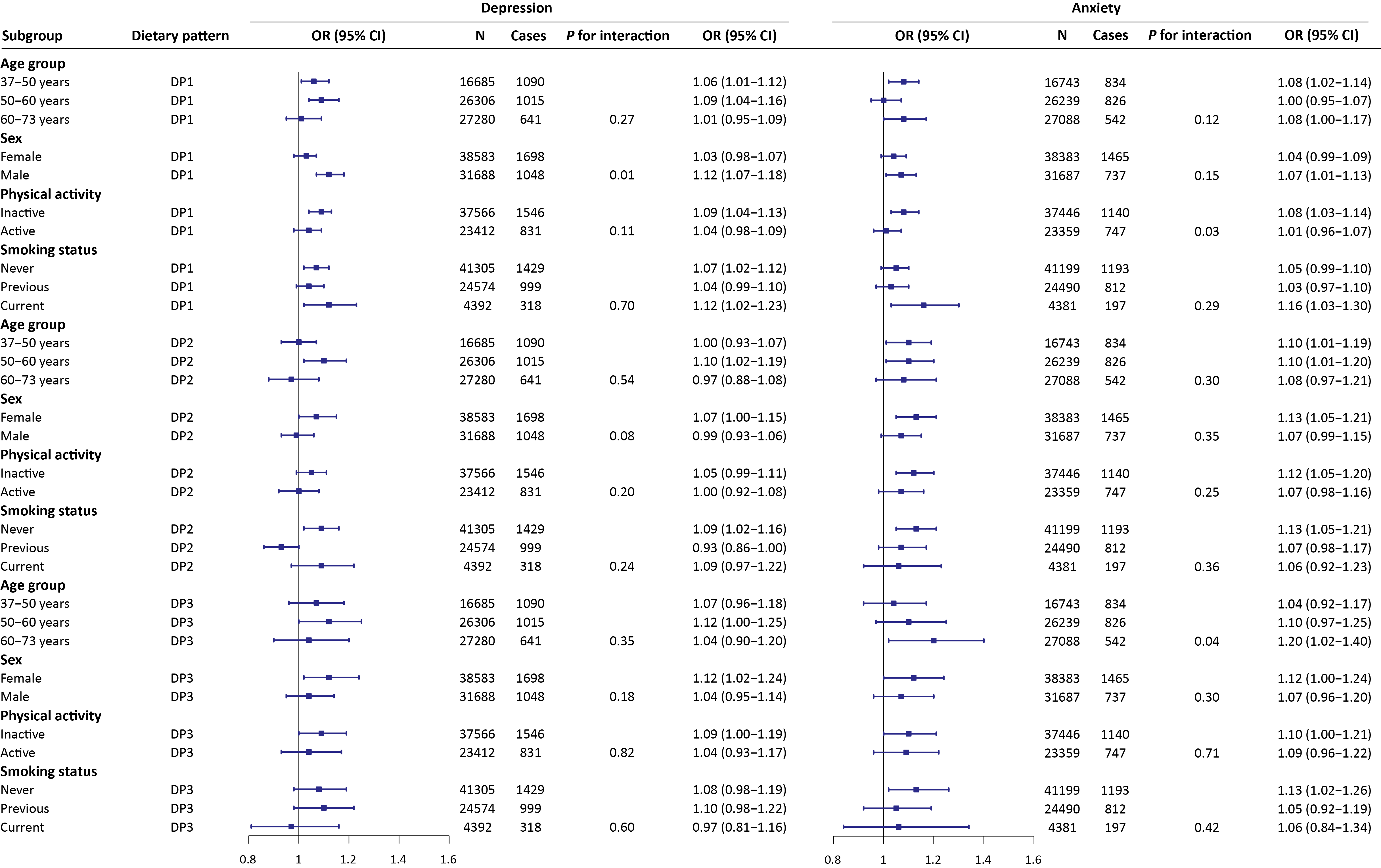
**

**Additional Figure S11** ORs (95% CIs) for associations between DPs and depressive and anxiety symptoms modified by risk factors.

All models were adjusted for age, sex, ethnicity, Townsend deprivation index, education level, smoking status, physical activity, history of hypertension, history of diabetes, and history of cardiovascular disease.
